# Prim-O-glucosylcimifugin ameliorates aging-impaired endogenous tendon regeneration by rejuvenating senescent tendon stem/progenitor cells

**DOI:** 10.1038/s41413-023-00288-3

**Published:** 2023-10-23

**Authors:** Yu Wang, Shanshan Jin, Dan Luo, Danqing He, Min Yu, Lisha Zhu, Zixin Li, Liyuan Chen, Chengye Ding, Xiaolan Wu, Tianhao Wu, Weiran Huang, Xuelin Zhao, Meng Xu, Zhengwei Xie, Yan Liu

**Affiliations:** 1 Laboratory of Biomimetic Nanomaterials, Department of Orthodontics, Peking University School and Hospital of Stomatology & National Center for Stomatology & National Clinical Research Center for Oral Diseases & National Engineering Laboratory for Digital and Material Technology of Stomatology & Beijing Key Laboratory of Digital Stomatology & Research Center of Engineering and Technology for Computerized Dentistry Ministry of Health & NMPA Key Laboratory for Dental Materials & Translational Research Center for Orocraniofacial Stem Cells and Systemic Health, Beijing, 100081 China; 2 CAS Center for Excellence in Nanoscience, Beijing Key Laboratory of Micro-nano Energy and Sensor, Beijing Institute of Nanoenergy and Nanosystems, Chinese Academy of Sciences, Beijing, 101400 China; 3 Peking University International Cancer Institute, Health Science Center, Peking University, Beijing, 100083 China; 4 Department of Orthopedics, the Fourth Medical Center of PLA General Hospital, Beijing, 100048 China

**Keywords:** Bone, Mitochondria

## Abstract

Adult tendon stem/progenitor cells (TSPCs) are essential for tendon maintenance, regeneration, and repair, yet they become susceptible to senescence with age, impairing the self-healing capacity of tendons. In this study, we employ a recently developed deep-learning-based efficacy prediction system to screen potential stemness-promoting and senescence-inhibiting drugs from natural products using the transcriptional signatures of stemness. The top-ranked candidate, prim-O-glucosylcimifugin (POG), a saposhnikovia root extract, could ameliorate TPSC senescent phenotypes caused by long-term passage and natural aging in rats and humans, as well as restore the self-renewal and proliferative capacities and tenogenic potential of aged TSPCs. In vivo, the systematic administration of POG or the local delivery of POG nanoparticles functionally rescued endogenous tendon regeneration and repair in aged rats to levels similar to those of normal animals. Mechanistically, POG protects TSPCs against functional impairment during both passage-induced and natural aging by simultaneously suppressing nuclear factor-κB and decreasing mTOR signaling with the induction of autophagy. Thus, the strategy of pharmacological intervention with the deep learning-predicted compound POG could rejuvenate aged TSPCs and improve the regenerative capacity of aged tendons.

## Introduction

Tendon rupture is a common injury in athletes and nonathletes alike, especially among elderly individuals, and is caused by high tensile burdens on tendons.^1–3^ Aging is characterized by the chronic and gradual impairment of functional capacities at various levels, including the cell, tissue, and organism levels, making the individual more susceptible to multiple diseases. Tendon aging is regarded as an inevitable degenerative process that results in impaired structural and functional conditions, such as decreased motor capacity, tendon rupture, and poor tendon healing.^4–6^ Despite the extensive literature on this topic, there is a large demand for clinical drugs capable of accelerating tendon rupture healing among aged populations.

Tendon homeostasis and repair are mainly dependent on the functions of tendon stem/progenitor cells (TSPCs),^7^ which are highly enriched and have robust tenogenic and self-renewal potential. Both stimuli from the extrinsic environment, such as inflammation, and intrinsic changes may contribute to TSPC senescence, which is characterized by the loss of stemness and tenogenic differentiation capacity. Tendon aging is accompanied by TSPC senescence and deteriorated tendon metabolism, both of which contribute to an impaired capacity for regeneration and repair.^8,9^ We thus speculated that the maintenance of stem cell stemness and the reduction in stem cell senescence are essential for tissue homeostasis and regeneration.

Both intrinsic (e.g., genetic regulation, signaling pathways, and organelles) and/or extrinsic parameters (e.g., cell culture microenvironment and biomaterials) can affect and regulate the self-renewal or differentiation of stem cells.^10–12^ Reprogramming via the genetic manipulation of transcription factors (Oct4, Sox2, Klf4, and Myc) and the application of exogenous signaling proteins have been used to promote stemness and maintain stem cell function.^13,14^ Artificial cellular microenvironments, including hypoxic/physiologic oxygen conditions and three-dimensional culture approaches (spheroids and cell sheets), have been reported to maintain cell cycle progression and enhance stemness. However, these strategies possess disadvantages, including low efficiency and quality, high costs, and mutation risk, which limit their application.^15–18^

Recently, natural small molecule drugs have emerged as a promising approach for rejuvenating senescent cells.^19,20^ However, selecting specific small molecules to both enhance stem cell stemness and reduce cellular senescence to enhance tissue regeneration in aged or diseased circumstances remains a challenge. Due to a lack of confirmed targets for stemness and aging, the transcriptional signature-based approach may be an excellent alternative with which to explore potential therapeutic strategies. Tendons at the neonatal stage present outstanding regenerative capacity that weakens over time.^21^ Given that most diseases are associated with changes in gene expression, we previously used Gene Ontology (GO) analysis and gene set enrichment analysis (GSEA) to determine that various types of crucial biological processes or signaling pathways changed from the neonatal to adult stage, indicating that differentially expressed genes (DEGs) between neonatal and adult tendons may be a reliable input source of gene signatures.^22^

To identify small molecule compounds that could specifically regulate stemness from transcriptional profiles, we recently developed a deep learning-based efficacy prediction system (DLEPS). This approach is able to extrapolate the empirical transcriptional data of known small molecules to numerous small molecules in silico. DLEPS has successfully predicted candidate molecules for highly diverse and complicated diseases based only on the gene signatures, leading to the discovery of several drugs that were effective in adipose browning, NASH, hyperuricemia, and aging.^23^ In this study, we first amended the analysis of the accuracy of DLEPS for each gene. Then, we evaluated the stemness score of each compound based on two scores: (i) Yamanaka factors and (ii) the DEGs of tendon tissues from neonatal rats compared with those from adult rats. From the top-ranked candidates in both scores, we identified prime-O-glucosylcimifugin (POG), a chromone extracted from *Saposhnikovia root*,^24^ which rejuvenated the phenotypes of senescent TSPCs in both in vitro long-term serial passaging and in vivo natural aging models. The systematic administration of POG or the local delivery of POG nanoparticles effectively promoted tendon healing in a partial transection tendon injury model. Mechanistically, POG simultaneously suppressed the nuclear factor-κB and mTOR signaling pathways, after which it induced autophagic activity through ATG7 to amplify the antisenescent effect of mTOR signaling. Finally, the combination of POG administration and biomimetic scaffold transplantation enhanced functional tendon regeneration in large-ranged, full-cut tendon window defects. These findings indicate that a single natural compound, namely, POG, is able to promote both stemness manipulation and rejuvenate senescence, ultimately rescuing the aging-impaired regeneration and repair capacity of tendons.

## Results

### A deep learning-predicted POG maintains rat TSPC stemness and functions

**Fig. 1:**
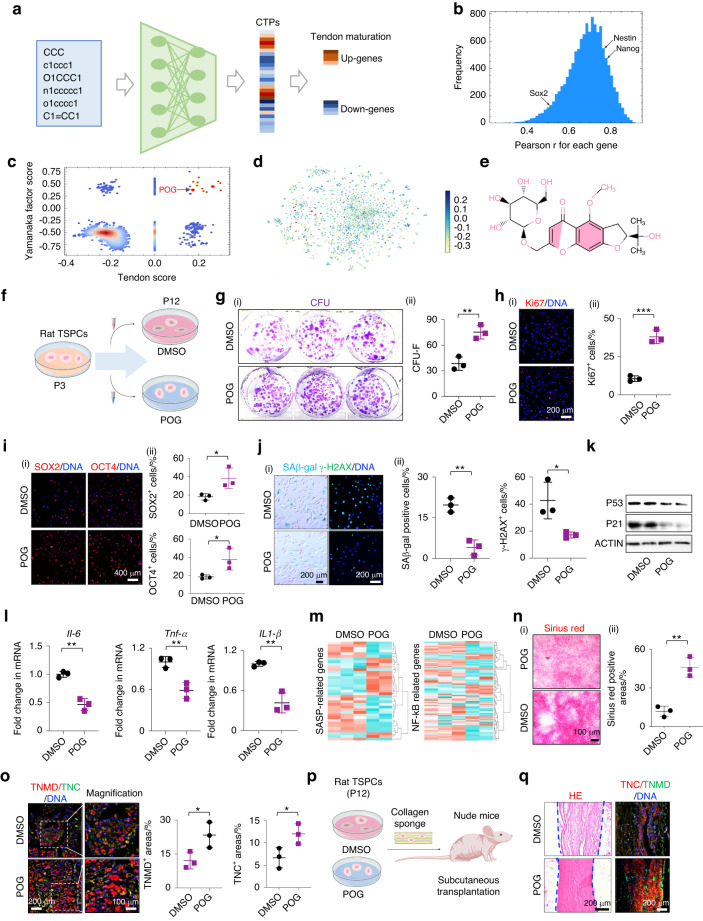
A deep learning-predicted POG maintains rTSPC stemness and functions. **a** Schematic of the deep neural network employed to predict stemness enhancement. **b** Distribution of Pearson correlation coefficient *r* of predicted and empirical profiles for all 12 328 genes over 3 000 molecules in the test set. **c** Scatter plot of the Yamanaka factor score versus the tendon score. **d** Highlight of positively predicted molecules in the t-SNE plot of the molecular library. **e** Schematic of the POG molecular formula. **f** Schematic of serial passaging of rTSPCs from 3-month-old rats treated with POG. **g** (i) CFU-F assay of rTSPCs at P12 with DMSO or POG treatment during serial passaging. (*n* = 3). **h** Immunofluorescence staining (i) and semiquantification (ii) of Ki67 in rTSPCs at P12 (*n* = 3). **i** Immunofluorescence staining of SOX2 and OCT4 in rTSPCs at P12 (*n* = 3). **j** (i) SAβ-gal staining (left panel) and immunofluorescence staining of the DNA injury-related protein γ-H2AX (right panel) in p12 rTSPCs (*n* = 3). **k** Western blotting of the senescence-related proteins P21 and P53 in rTSPCs at P12. **l** RT‒qPCR of *Il-6* and *Tnf-a* gene expression in rTSPCs at P12 (*n* = 3). **m** Heatmap of the differentially expressed gene profiles of rTSPCs at P12 *n* = 3. **n** (i) Sirius Red staining of rTSPCs at P12 (*n* = 3). **o** Immunofluorescence staining of the tenogenic markers TNMD and TNC in rTSPCs at P12 *(n* = 3). **p** Schematic of subcutaneous transplantation of collagen sponges with rTSPCs at P12. **q** HE and immunofluorescence staining of the tenogenic markers TNC and TNMD *n* = 5. Data are represented as the mean ± SD. (*P < 0.05; **P < 0.01; ***P < 0.001)

In search of specific small molecules that could restore the age-related decline in tendon healing capacity, we employed DLEPS to predict stemness-targeting small molecules. Basically, DLEPS utilizes a two-step approach to predict the pharmacological effects of small molecules. DLEPS first applies deep neural networks to predict the quantitative relationship between small molecules and gene expression changes in cells after small molecule treatment. DLEPS then uses the GSEA algorithm to evaluate whether the gene expression changes caused by small molecules reverse disease-associated gene expression changes to predict the pharmacological effects of small molecules. The utility of DLEPS has been demonstrated across various diseases, including inducing adipose browning, treating NASH and hyperuricemia, and reversing aging phenotypes.^23^ Compared with the latest version of DLEPS, we upgraded the statistical analysis of how well each gene was predicted in all 12,328 genes using the Pearson correlation coefficient (Fig. 1a, b). In neonatal tendons, TSPCs are highly enriched and have robust tenogenic and self-renewal potentials.^21,22^ Very recently, we generated two groups of expression signatures from neonatal and adult rats and found a distinct decline in the stemness and regenerative capacity of rat TSPCs (rTSPCs) from the neonatal to adult stage.^22^ Here, we employed two groups of gene signatures to calculate the efficacy scores, namely, a Yamanaka factor score defined by four Yamanaka factors and a tendon score, using the top-ranked DEGs from a comparison of the transcription of tendon tissue from neonatal rats and adult rats (Fig. 1c). Finally, we selected the intersection of top-ranked candidates in both scores (12 molecules in total) to be tested in the cell culture or directly in the animal models (Fig. 1d, Table. S1). POG, a chromone extracted from *Saposhnikovia* root, was top ranked with good pharmacological properties (Fig. 1e). Although studies have demonstrated the functional roles of POG as an anticonvulsant, anti-inflammatory, and anticancer molecule,^24,25^ reports on its activities in the regulation of aging-related stem cell functions and tissue maintenance are currently lacking.

To determine whether POG rescues the phenotypic characteristics of rTSPCs, we first adopted a replicative cellular senescence model, which has been widely utilized to investigate cell stemness and senescence.^26–28^ Flow cytometry showed that rTSPCs were positive for the mesenchymal stem cell markers CD105, CD90, and C29 and negative for CD34 (a hematopoietic stem cell marker) and CD45 (a leukocyte marker) (Fig. S1a). Our preliminary experiments also confirmed that replicative senescence caused by serial passaging from passage 3 (P3) to passage 12 (P12) disrupted the stemness and differentiation capacities of rTSPCs isolated from 3-month-old (3M) rats (young rats; Fig. S2a–h). Next, according to previous studies and preliminary experiments,^29^ POG at a relatively moderate concentration of 20 μmol·L^−1^ displayed a stemness-promoting effect, which was reflected by RT‒qPCR results (Fig. S1b). Therefore, in follow-up experiments, 20 μmol·L^−1^ POG was added to the culture medium of rTSPCs in each passage from P3 to P12. An equal amount of the vehicle control dimethyl sulfoxide (DMSO) was added to the control group to exclude the influence of solvent (Fig. 1f). Compared with the DMSO-treated cells, the POG-treated rTSPCs showed markedly elevated self-renewal and proliferation, as evidenced by the larger numbers of colonies and a marked increase in Ki67^+^ cells (Fig. 1g, h). Immunofluorescence staining and RT‒qPCR demonstrated that POG treatment significantly enhanced the expression of SOX2 and OCT4 in rTSPCs at P12, indicating that POG helps to maintain rTSPC stemness during long-term passage (Fig. 1i, S3a). Moreover, POG treatment ameliorated multiple aging hallmarks, as evidenced by the following findings: (i) significantly decreased SAβ-gal-positive cells; (ii) repressed DNA damage response, as indicated by fewer γ-H2AX^+^ cells (Fig. 1j); (iii) markedly reduced expression of the aging-associated factors P53 and P21 (Fig. 1k); and (iv) reduced mRNA expression of *Il-6*, *Il-1β*, and *Tnf-α*, which indicated a decline in SASP (Fig. 1l). Consistent with these results, microarray also indicated that the expression of SASP-related genes was significantly decreased in the POG-treated rTSPCs at P12. Additionally, GSEA showed that POG-downregulated genes were associated with the TNF-α/NF-κB signaling pathway, further confirming the profound effect in delaying senescence (Fig. 1m).

Next, we evaluated whether sustained exposure to POG helped to maintain the tenogenic differentiation potential of rTSPCs after prolonged passages. Before tenogenic induction, we stopped POG treatment in rTSPCs at P12 and then transferred the DMSO- and POG-treated passaged rTSPCs into tenogenic differentiation medium without POG. As a result, the POG-treated passaged rTSPCs showed strong Sirius Red staining after 14 d of tenogenic induction, as well as marked increases in the protein expression of the key tenogenic markers TNMD, TNC, scleraxis (SCX), and fibromodulin (FMOD), compared with the DMSO-treated passaged rTSPCs, indicative of the functional rejuvenation of senescent rTSPCs (Fig. 1n, o, S3b). Furthermore, we subcutaneously transplanted DMSO- and POG-treated passaged rTSPCs at P12 with collagen sponges into nude mice and harvested tissues 8 weeks after surgery. Hematoxylin and eosin (HE) and immunofluorescence staining showed that POG-treated passaged rTSPCs maintained their intrinsic regenerative capacity, allowing the formation of dense tendon collagen fibers with higher levels of TNC and TNMD expression (Fig. 1p, q).

The spheroid-forming capacity of stem cells could represent their own stemness property.^30,31^ Using our previously reported methods,^22^ we constructed multicellular spheroids of DMSO- and POG-treated rTSPCs at P12 by replacing standard culture dishes with low-adhesion culture dishes for a 7-d culture period (Fig. S3c). Long-term passage led to a poor spheroid-forming capacity in the DMSO-treated passaged rTSPCs (~6.0 ± 4.0 total); in contrast, the POG-treated passaged rTSPCs were found to assemble into a higher number of spheroids (~38.5 ± 6.5 total) (Fig. S3d). Immunofluorescence analysis demonstrated that spheroids formed by the POG-treated passaged rTSPCs exhibited higher expression levels of SOX2 colocalized with OCT4 (Fig. S3e). Next, the tenogenic differentiation capacity of these spheroids was evaluated by transferring spheroids onto normal-adhesion culture dishes coated with Matrigel supplemented with the tenogenic induction medium for 14 d. Scanning electron microscopy (SEM) revealed that spheroids formed by the POG-treated passaged rTSPCs stretched out more longitudinally aligned collagen fibers (Fig. S3f). Immunofluorescence analysis confirmed that the POG-restored passaged rTSPCs expressed the tendon-specific factor TNMD at higher levels after 21 d of induction (Fig. S3g). Collectively, these findings indicate that pharmacological manipulation with POG during in vitro long-term passage rescued the intrinsic tenogenic differentiation potential of rTSPCs by both maintaining stemness and inhibiting senescence.

### POG nanoparticles rescue aged tendon self-healing capacity by enhancing TSPC stemness and rejuvenating senescent phenotypes

**Fig. 2:**
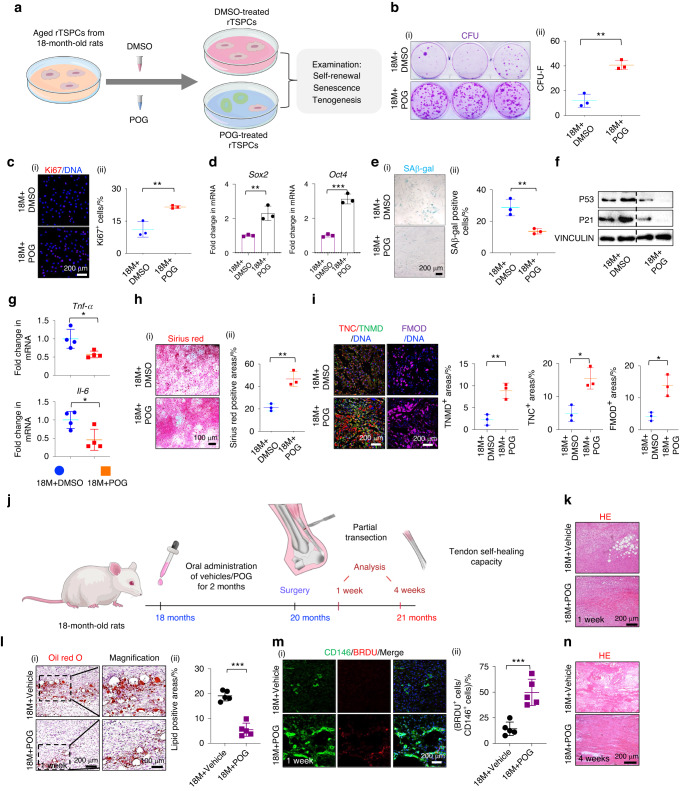
Systemic delivery of POG rescues the aged tendon self-healing capacity by enhancing rTSPC stemness and rejuvenating senescent phenotypes. **a** Schematic outlining the design of POG intervention in senescent rTSPCs from aged rats. **b** CFU-F assay of DMSO- and POG-treated aged rTSPCs (*n* = 3). **c** Immunofluorescence staining of Ki67 in DMSO- and POG-treated aged rTSPCs (*n* = 3). **d** RT‒qPCR of the stemness proteins SOX2 and OCT4 in DMSO- and POG-treated aged rTSPCs (*n* = 3). **e** SAβ-gal staining of DMSO- and POG-treated aged rTSPCs (*n* = 3). **f** Western blotting of senescence-related proteins P53 and P21 in DMSO- and POG-treated aged rTSPCs (*n* = 3). **g** RT‒qPCR of *Il-6* and *Tnf-a* gene expression in DMSO- and POG-treated aged rTSPCs (*n* = 4). **h** (i) Sirius Red staining of DMSO- and POG-treated aged rTSPCs (*n* = 3). **i** (i) Immunofluorescence staining of the tenogenic markers TNC, TNMD and FMOD in DMSO- and POG-treated aged rTSPCs (*n* = 3). **j** Schematic outlining systematic administration of POG on the partial transection tendon injury in 18-month-old rats. **k** HE staining of injured Achilles tendons *n* = 5. **l** (i) Oil Red O staining of injured Achilles tendons (*n* = 5). **m** (i) Immunofluorescence costaining of CD146 and BRDU in injured Achilles tendons (*n* = 5). **n** HE staining of injured Achilles tendons *n* = 5. Data are represented as the mean ± SD. (*P < 0.05; **P < 0.01; ***P < 0.001)

Given the beneficial therapeutic effects of POG on the in vitro replicative senescence model of rTSPCs, we next investigated whether POG treatment could directly rescue impaired biological functions of rTSPCs induced by in vivo natural aging. First, tendons from 18-month-old (18M) rats (aged rats) displayed typical aging characteristics, including collagen degradation, more lipid deposition in TEM images and expression of the senescence markers P21 and γ-H2AX (Fig. S4a–c). Second, tendon injury in 18-month-old aged rats displayed increased lipid deposition, accumulation of senescent cells and secretion of inflammatory factors compared to that of 3-month-old rats (Fig. S4d–k). Third, rTSPCs isolated from aged rats displayed seriously impaired colony-forming, cell proliferation, and tenogenic differentiation capacities, as demonstrated by a larger number of SAβ-gal-positive cells, higher levels of P53 and P21 expression, and elevated mRNA expression of *Il-6* and *Tnf-α* (Fig. S5a–f). These results indicate that the 18M aged rat model is a reliable animal model to investigate the antiaging functions of POG. Then, aged rTSPCs isolated from the aged rats were treated with 20 μmol·L^−1^ POG (18M + POG group) or DMSO (18M + DMSO group) as a control once every 2 d for 7 d (Fig. 2a). CFU-F assays showed that POG treatment markedly increased the number of CFU-Fs in aged rTSPCs compared to that in the 18M + DMSO group (Fig. 2b). In vitro serial passaging-induced cell senescence is significantly distinct from in vivo natural aging-induced senescence, which may explain some differences between the results here and those in Fig. 1g. RT‒qPCR and immunofluorescence staining revealed that POG treatment significantly enhanced the protein expression of Ki67, SOX2, and OCT4 in aged rTSPCs (Fig. 2c, d). In addition, the percentage of SA-β-gal-positive senescent cells was markedly decreased in the 18M + POG group compared to the 18M + DMSO group (Fig. 2e). This finding was coupled with a significant suppression of the senescence-related proteins P21 and P53 (Fig. 2f). POG treatment also decreased the gene expression of several SASP factors, including *Tnf-α* and *Il-6*, in aged rTSPCs (Fig. 2g).

Prior to tenogenic induction, we stimulated the senescent rTSPCs with POG once every 2 d for 5–7 d before transferring them into differentiation medium for 10–14 d. Immunofluorescence showed that the senescent TSPCs from the 18M + POG group possessed markedly increased positive areas of Sirius Red staining and higher expression levels of the key tenogenic markers TNC, TNMD, and FMOD than those from the 18M + DMSO group (Fig. 2h, i). Combining the results from the in vitro serial passage-inducing senescence model with the in vivo natural aging model, we found that the DLEPS screening system could efficiently and precisely identify a small-molecule compound, POG, that maintains rTSPC stemness and inhibits senescence.

Next, we examined the potential of the systemic delivery of POG to improve endogenous rTSPC functions and self-healing capacity in aged rats (Fig. 2j). The aged rats were first orally administered POG (18M + POG group) or DMSO as a vehicle (18M+vehicle group) once a day for 2 month. Oral administration ended 2 d prior to the surgery. Then, a partial incision was made in the Achilles tendon, as previously described.^22^ After 7 d, the injured tendons from the 18M + POG group displayed reparative effects that were comparable to those in the injured tendons from the young rats (Fig. S4d–f), including relatively complete tendon structure with continuous thin fibers and a significant reduction in lipid droplets and fatty-acid-binding protein 4 (FABP4)-positive adipose cells (Fig. 2k, l). Moreover, a greater percentage of CD146^+^BrdU^+^ endogenous rTSPCs accumulated at sites of injury in the 18M + POG group than in the 18M+vehicle group (Fig. 2m). After 4 weeks, the injured tendons with POG pretreatment showed more ordered and thicker collagen and reduced inflammatory infiltration, as evidenced by the significantly decreased proportion of IL-6^+^ and MMP9^+^ cells, while fragmentary fibers and persistent CD68^+^ inflammatory cells were observed in injured areas in the 18M+vehicle group (Fig. 2n, S6a, b). Moreover, despite the existence of a small gap in reparative histological effects compared to those of uninjured tendons from young rats (Fig. S4a), two months of POG pretreatment was found to delay the progression of collagen disorder and relieve cellular senescence in aged, uninjured tendons, as evidenced by the declining ratio of γ-H2AX- and P21-positive cells and immunofluorescence staining (Fig. S6c–e). Hence, the oral administration of POG effectively relieved multiple phenomena of tendon aging, including cellular senescence, lipid droplet deposition, inflammatory infiltration, and impaired recruitment capacity of endogenous stem cells.

**Fig. 3:**
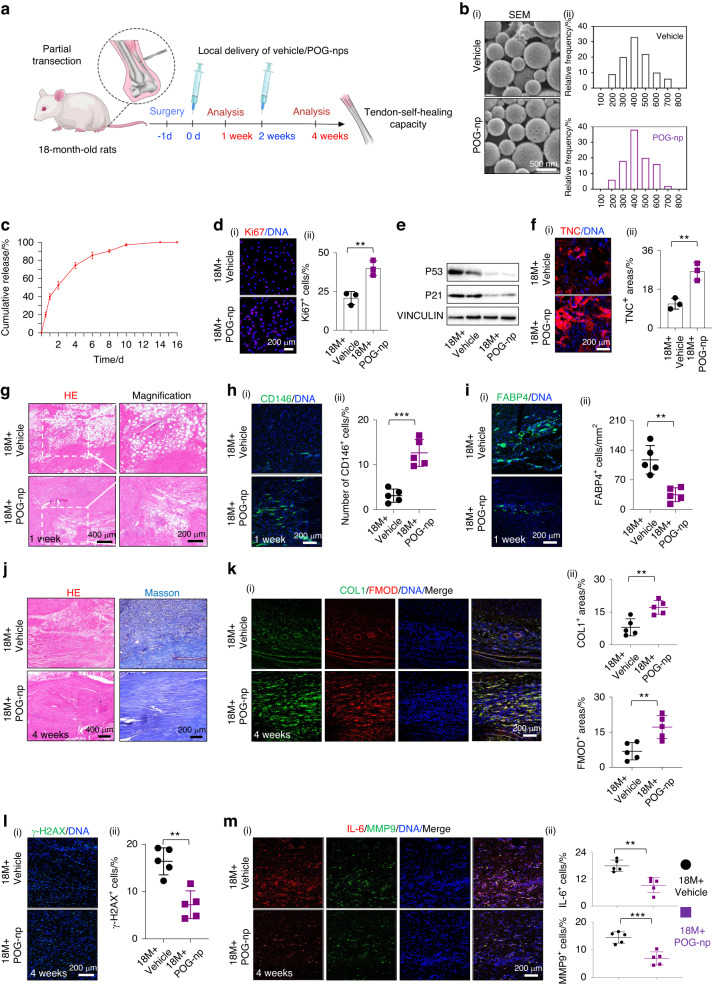
Local delivery of POG nanoparticles effectively promotes endogenous tendon healing by inhibiting lipid droplet deposition and inflammatory progression. **a** Schematic outlining local delivery of POG-nps to partial transection tendon injuries in aged rats. **b** (i) SEM images of PLGA (vehicle) and PLGA nanoparticles loaded with POG (POG-nps). (ii) Size distribution determined by dynamic light scattering (*n* = 4). **c** Cumulative release curves of POG from the POG-nps obtained using HPLC‒MS analysis. **d** (i) Immunofluorescence staining of Ki67 (*n* = 3). **e** Western blotting of the senescence-related proteins P53 and P21 (*n* = 3). **f** (i) Immunofluorescence staining of the tenogenic marker TNC (*n* = 3). **g** HE staining of neotendons from the aged rats *n* = 5. **h** (i) Immunofluorescence staining of CD146 in sections (*n* = 5). **i** Immunofluorescence staining of FABP4 (*n* = 5). **j** HE and Masson trichrome staining of neotendons *n* = 5. **k** (i) Immunofluorescence staining of COL1 and FMOD (*n* = 5). **l** (i) Immunofluorescence staining of γ-H2AX (*n* = 5). **m** (i) Immunofluorescence staining of IL-6 and MMP9 (*n* = 5). Data are represented as the mean ± SD. (**P < 0.01; ***P < 0.001)

To provide a local sustained release of POG at the sites of tendon injury, we applied FDA-approved polylactic glycolic acid (PLGA) nanoparticles to load POG (POG-nps; Fig. 3a). SEM showed that both POG-nps and PLGA nanoparticles were spherically shaped, with similar diameters of 100–300 nm in the dry state (Fig. 3b). POG was gradually released over a 14-d period, as determined by high-performance liquid chromatography (HPLC‒MS; Fig. 3c). To determine whether the drug-releasing particles had antisenescent and protenogenic functions, we treated aged rTSPCs from aged rats with POG-nps at a concentration of 20 μmol·L^−1^ after dilution that was consistent with the previous dosage in vitro. POG-nps effectively rescued aged rTSPC functions, as evidenced by increased Ki67^+^ cells and reduced protein expression levels of P53 and P21 in aged rTSPCs in vitro (Fig. 3d, e). Moreover, pretreatment with POG-nps for 4 d prior to tenogenic induction promoted the expression of the tenogenic markers FMOD and TNC, with reduced activation of P65 and decreased secretion of the inflammatory factor IL-6 in aged rTSPCs (Fig. 3f, S7a, b), thereby recapitulating the antisenescent effect of POG.

To further verify the in vivo effect of the local delivery of drug-loaded nanoparticles on small-range tendon repair in aged rats, we injected POG-nps (18M + POG-nps) or PLGA nanoparticles alone as a vehicle (18M+vehicle) into the partially transected Achilles tendons at Days 0 and 7. Fluorescence staining showed retention of Cy5 in the tendon tissue at Days 0 and 7, indicating that the PLGA nanoparticles were an effective drug delivery vehicle in injured tendons (Fig. S7c). Similar to the therapeutic effects of the oral administration of POG (Fig. 2k–n), the local injection of POG-nps largely attenuated the formation of vacuole-like structures and lipid droplet deposition and substantially recruited larger amounts of endogenous CD146^+^ cells to the injured tendons after 7 d compared to those of the 18M+vehicle group (Fig. 3g–i). Four weeks after the incision injury, densely packed collagen fibers formed, with higher expression levels of the tenogenic markers COL1 and FMOD in the 18M + POG-nps group, indicating that POG-nps maintained the tenogenic differentiation of endogenous rTSPCs (Fig. 3j, k). Moreover, the delivery of POG-nps alleviated the expression of the senescence marker γ-H2AX (Fig. 3l) and the inflammatory markers MMP9 and IL-6, which are characteristics of age-related tendon diseases (Fig. 3m).

Both the systemic administration of POG and the local delivery of POG nanoparticles effectively enhanced the healing capacity of partial transection in aged rat tendons by enhancing rTSPC stemness, rejuvenating senescent phenotypes, and simultaneously suppressing lipid droplet deposition and inflammatory progression.

### POG prevents rTSPC senescence by suppressing NF-κB

**Fig. 4:**
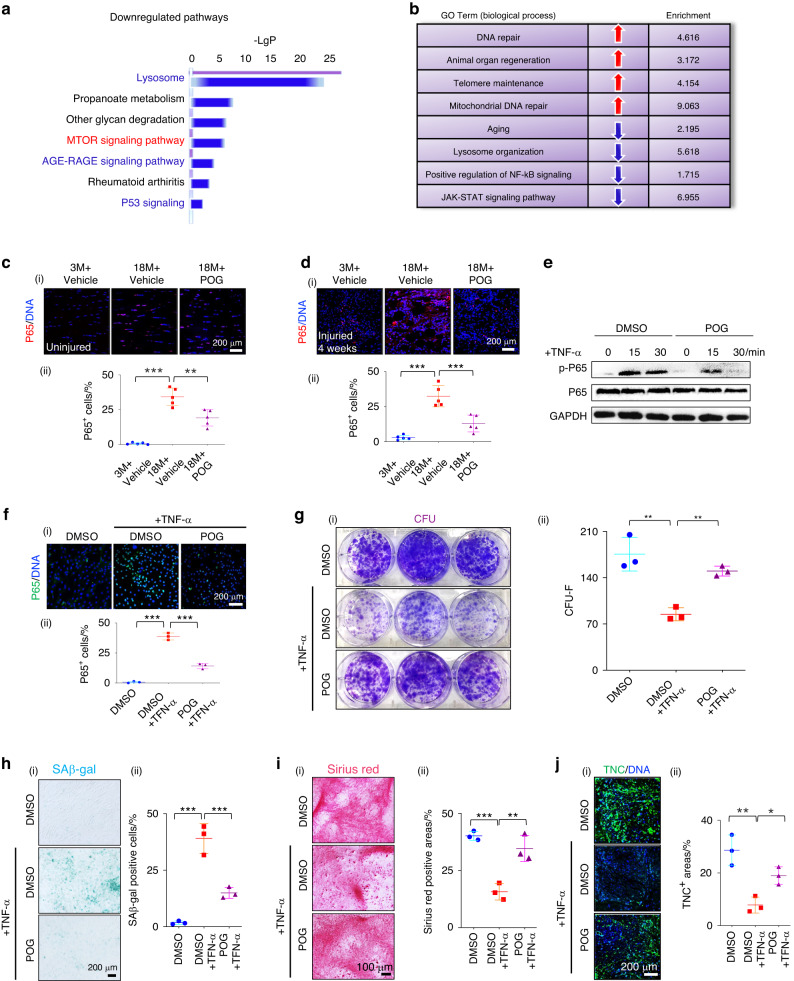
POG prevents rTSPC senescence by suppressing NF-κB signaling. **a** Gene set enrichment analysis of downregulated pathways of aged rTSPCs isolated from aged rats with or without POG treatment. **b** GO term analysis of related biological processes of aged rTSPCs isolated from aged rats with or without POG treatment. **c** (i) Immunofluorescence staining of P65 in uninjured tendons (*n* = 5). **d** (i) Immunofluorescence staining of P65 in injured tendons (*n* = 5). **e** Western blotting of the phosphorylation of P65. **f** (i) Immunofluorescence staining of P65. (ii) Semiquantification of (i) (*n* = 3). **g** (i) CFU-F assay of DMSO- and POG-treated rTSPCs from young rats after 14 d of TNF-α stimulation (*n* = 3). **h** (i) SAβ-gal staining of DMSO- and POG-treated rTSPCs from young rats after 14 d of TNF-α stimulation (*n* = 3). **i** (i) Sirius Red staining of DMSO- and POG-treated rTSPCs from young rats after 14 d of TNF-α stimulation. (ii) Semiquantification of (i) (*n* = 3). **j** (i) Immunofluorescence staining of TNC in DMSO- and POG-treated rTSPCs from young rats after 14 d of TNF-α stimulation. (ii) Semiquantification of (i) (*n* = 3). Data are represented as the mean ± SD. (*P < 0.05; **P < 0.01; ***P < 0.001)

To explore how POG prevents aged rTSPC senescence, we first performed RNA sequencing (RNA-seq) to compare the transcriptome profiles of senescent rTSPCs from aged rats treated with POG or DMSO once every 2 d for 7 d. GO enrichment and pathway enrichment analysis demonstrated that aging-related genes and several aging-related signaling pathways, including p53 and AGE-RAGE signaling, were decreased in the POG-treated aged rTSPCs compared to the DMSO-treated aged rTSPCs (Fig. 4a). Moreover, multiple beneficial biological processes (e.g., animal organ regeneration, telomere maintenance, and mitochondrial DNA repair) exhibited an elevated tendency, indicative of the functional recovery of POG-treated aged rTSPCs (Fig. 4b). Notably, a consistent downregulation was observed in senescence-associated pathways, including NF-κB and mTOR, which have been previously reported to regulate the senescence of various adult stem cells.^32,33^ Recent studies have shown that the inhibition of mTOR or NF-κB could reduce inflammation or promote tendon repair.^34,35^ However, it was unclear whether the NF-κB pathway was also overactivated in the progression of aged tendons or TSPC senescence. Therefore, we performed immunofluorescence staining of the active form of the NF-κB subunit p65 on uninjured tendon sections from young and aged rats, which has been widely used as an indicator of the activation of the NF-κB pathway.^36–38^ The results revealed that the uninjured tendons from the aged rats had a higher ratio of cells expressing p65 than those from the young rats, and POG administration markedly reduced the percentage of p65^+^ cells in the aged rats (Fig. 4c). After tendon injury, different types of cells begin to expand or are recruited to the injured areas.^39^ NF-κB is often activated in response to injury stimuli and is beneficial for the start-up of the body response in the early phase. However, the chronic activation of NF-κB could delay tissue healing.^40^ Four weeks after the incision injury, we found that distinct p65-positive expression was sustained at the injury sites of aged rats compared to young rats, after which the administration of POG markedly repressed its expression (Fig. 4d). NF-κB can be activated by proinflammatory cytokines, such as TNF-α and IL-17, in inflammatory diseases and tissue injuries.^41^ Moreover, chronic inflammatory insult could induce cell senescence in vivo.^42^ To determine whether POG could inhibit TNF-induced NF-κB in rTSPCs isolated from young rats, we continuously stimulated rTSPCs treated with POG or DMSO with 10 ng·mL^−1^ TNF-α for 0, 15, and 30 min. As shown in Fig. 4e, POG inhibited TNF-α-induced p65 phosphorylation in rTSPCs. Immunofluorescence staining confirmed that POG inhibited p65 activation in the POG-pretreated rTSPCs compared to the DMSO-pretreated rTSPCs (Fig. 4f). Colony-forming experiments revealed that POG pretreatment once every 2 d for 14 d largely rescued the impaired CFU-F formation capacity that was caused by persistent TNF-α stimulation (Fig. 4g). Notably, TNF-α stimulation induced distinct cellular senescence, which was significantly suppressed by POG treatment, as evidenced by the results of SA-β-gal staining (Fig. 4h). Previous studies showed that chronic inflammation could induce senescence in mesenchymal stem cells, which may explain the poor tissue healing occurring in aging and the chronic disease of multiple tissues.^43,44^ Sirius Red staining showed that POG treatment significantly attenuated the inhibitory effect of TNF-α on collagen matrix formation by rTSPCs (Fig. 4i). Furthermore, immunofluorescence staining revealed that POG treatment significantly reduced the inhibitory effect of TNF-α on TNC secretion (Fig. 4j).

### POG maintains aged rTSPC function by inhibiting mTOR signaling and activating autophagy

**Fig. 5:**
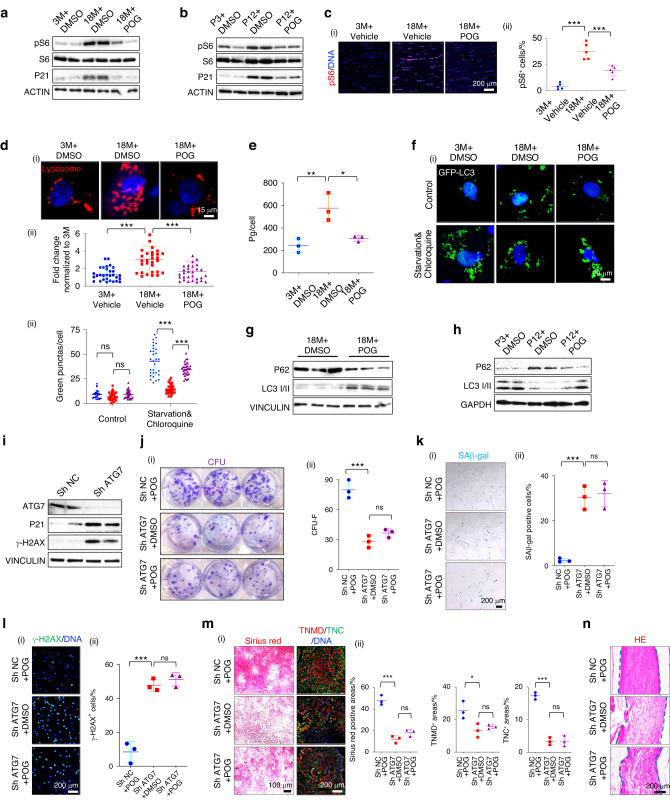
POG maintains aged rTSPC functions by inhibiting mTOR signaling and activating autophagy. **a** Western blotting of pS6 and P21 in rTSPCs. **b** Western blotting of pS6 and P21 in rTSPCs. **c** (i) Immunofluorescence staining of pS6 in uninjured tendons (*n* = 5). **d** (i) LysoTracker staining showing lysosomes of TSPCs. (ii) Semiquantification of (i). (*n* = 30 different cells). **e** Total intracellular protein content of rTSPCs (*n* = 3). **f** (i) Immunofluorescence staining of the autophagy protein LC3 in rTSPCs. (ii) Semiquantification of (i) (*n* = 30 different cells). **g** Western blotting of LC3I/II and P62 in rTSPCs. **h** Western blotting of the autophagy proteins LC3I/II and P62 in rTSPCs. **i** Western blotting of ATG7, P21 and γ-H2AX in rTSPCs *n* = 3. **j** (i) CFU-F assay of rTSPCs from aged rats. (ii) Semiquantification of (i) (*n* = 3). **k** (i) SAβ-gal staining of rTSPCs. (ii) Semiquantification of (i) (*n* = 3). **l** (i) Immunofluorescence staining of the DNA injury-related protein γ-H2AX in rTSPCs. (ii) Semiquantification of (i) (*n* = 3). **m** (i) Sirius Red staining (left panel) and immunofluorescence staining (right panel) of the tenogenic markers TNC and TNMD. (ii) Semiquantification of (i) (*n* = 3). **n** HE staining of parallel-aligned collagen structures *n* = 3. Data are represented as the mean ± SD. (ns: not significant; *P < 0.05; **P < 0.01; ***P < 0.001)

Given that the inhibition of the mTOR pathway promotes lifespan and its role in the aging of adult stem cells,^45,46^ we sought to investigate whether POG also inhibited rTSPC senescence during in vitro serial passaging and in vivo natural aging by inhibiting the activation of mTOR. Senescent rTSPCs from the aged rats with POG stimulation (18M + POG) showed inhibited phosphorylated S6 (pS6) expression compared to the DMSO-treated controls (18M + DMSO) (Fig. 5a). Similarly, the POG-treated passaged rTSPCs at P12 (P12 + POG) displayed distinctly reduced S6 phosphorylation compared to the DMSO-treated passaged rTSPCs from the young rats at P12 (P12 + DMSO) (Fig. 5b). The immunofluorescence of uninjured tendon sections in the 18M + POG group showed significantly lower levels of pS6 expression than those in the 18 M+vehicle group (Fig. 5c). As reported previously, mTOR is a negative regulator of autophagy, and its activities are gradually increased in multiple tissues during aging. The utility of mTOR inhibition as an autophagy-inducing therapy for age-related diseases has been explored widely.^47,48^ The slow rate of autophagy and the accumulation of autophagic vesicles and lysosomes are characteristics of senescent phenotypes.^49^ Here, we visualized lysosomal trafficking using LysoTracker and found that aged rTSPCs from the 18+ POG group displayed markedly decreased lysosomes, close to the normal level observed in young rTSPCs from young rats (3M + DMSO) (Fig. 5d). Senescent cells were often ineffective in eliminating abnormal proteins,^50,51^ as reflected by the increased protein content in aged rTSPCs from the 18 + DMSO group and its recovery to normal levels in aged rTSPCs after POG treatment (Fig. 5e). In fact, S-βgal is an intracellular lysosomal enzyme. When stem cells become senescent, excessive staining of S-βgal will occur in the cells, which also reflects the abnormal metabolic condition of lysosomes.^52^ Therefore, our results demonstrate that POG is able to improve the condition of lysosomes in senescent cells. Next, we examined whether POG treatment could restore normal autophagic flux in aged rTSPCs from aged rats. Immunofluorescence staining of lysosomes and microscopic detection of cytoplasmic GFP-LC3 dots revealed that POG induced autophagic flux in aged rTSPCs (Fig. 5f). Furthermore, supplementation with POG significantly reduced the p62 protein level and increased the microtubule-associated protein 1 A/1B light chain 3B (LCB II) expression level in aged rTSPCs (Fig. 5g). Similarly, serial passaging also increased the expression of the typical autophagy target protein SQSTM1/p62 and decreased the LCB II level, which was reversed by POG treatment (Fig. 5h). Notably, P62 can serve as a receptor for vesicles to be degraded by autophagy, as well as for ubiquitinated protein aggregates to be cleared. The p62 protein can thus target autophagosomes and promote the clearance of ubiquitinated proteins.^53^ Therefore, the reduction in P62 protein levels here also reflects the reduction in abnormal proteins to some extent.

These results indicate that senescent rTSPCs induced by in vitro serial passaging and in vivo natural aging displayed elevated phosphorylated S6 expression and impaired autophagic activity, which could both be reversed by in vitro POG treatment. This finding further confirms that the in vitro long-term passage model was also a reliable cell senescence model that could partially reflect the functional changes in natural aging cells.

*ATG5, ATG7*, and *BECN1* (encoding Beclin-1) code for essential components of the autophagic machinery and are downregulated during normal aging.^47,54^ We thus hypothesized that the beneficial biological effects of POG on aged rTSPCs from 18-month-old rats depend on autophagic regulation. For analysis of this hypothesis, aged rTSPCs were stably infected with a lentiviral vector expressing shRNA targeting the autophagy gene ATG7 (*sh-ATG7*). Western blotting showed that the expression of P21 and γ-H2AX was significantly elevated after the knockdown of ATG7 (Fig. 5i). Importantly, ATG7 knockdown abrogated the functions of POG in maintaining the colony-forming capacity of aged rTSPCs (Fig. 5j), inhibiting cellular senescence (Fig. 5k, l) and rescuing tenogenic differential potential during in vitro serial passage (Fig. 5m, n). Taken together, these findings indicate that autophagic induction is indeed necessary for the therapeutic effects of POG on the functions of senescent rTSPCs.

### POG treatment rescues middle-aged human TSPC stemness and regenerative capacity by activating autophagy

**Fig. 6:**
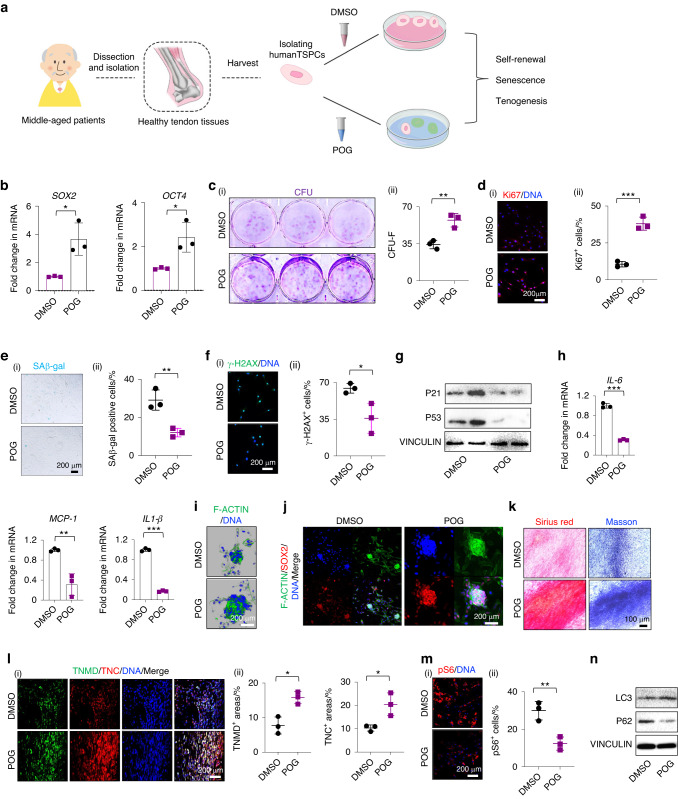
POG treatment ameliorates senescent phenotypes from senescent hTSPCs by maintaining stemness and inhibiting senescence. **a** Schematic outlining of human tendon sample collection, isolation of human TSPCs (hTSPCs) and POG treatment in hTSPCs. **b** RT‒qPCR of SOX2 and OCT4 (*n* = 3). **c** (i) CFU-F assay of DMSO- and POG-treated senescent hTSPCs. (ii) Semiquantification of (i) (*n* = 3). **d** (i) Immunofluorescence staining of Ki67. (ii) Semiquantification of (i) (*n* = 3). **e** (i) SAβ-gal staining of DMSO- and POG-treated senescent hTSPCs. (ii) Semiquantification of (i) (*n* = 3). Immunofluorescence staining of spheroids (*n* = 3). **f** (i) Immunofluorescence staining of γ-H2AX. (ii) Semiquantification of (i) (*n* = 3) **g** Western blotting of P21 and P53 (*n* = 3). **h** RT‒qPCR of *IL-6*, *MCP-1*, and *IL-1β* (*n* = 3). **i** Immunofluorescence staining of spheroids (*n* = 3). **j** Immunofluorescence staining of F-actin and SOX2 in spheroids. **k** Sirius Red and Masson’s trichrome staining. **l** Immunofluorescence staining of the tenogenic markers TNMD and TNC. **m** (i) Immunofluorescence staining of pS6. (ii) Semiquantification of (i) (*n* = 3). **n** Western blotting of the autophagy proteins LC3I/II and P62 (*n* = 3). Data are represented as the mean ± SD. (*P < 0.05; **P < 0.01; ***P < 0.001)

Given the beneficial effects of POG on stemness enhancement, namely, the inhibition of cellular senescence and the maintenance of tenogenic differentiation capacity in senescent rTSPCs from both in vitro serial passaging and in vivo natural aging, we next investigated whether POG could intervene in the senescent phenotypes of human TSPCs (hTSPCs), providing further evidence of its potential use for the treatment of age-related tendon diseases in humans. In the context of obtaining consent from potential study subjects, it was difficult to obtain tendons from individuals who were too elderly. Here, we collected healthy tendons from middle-aged patients (approximately 50 years old) to isolate hTSPCs as our subsequent experimental model (Fig. 6a). Existing studies have also demonstrated that human TSPCs from donors with an average age of 63 ± 14 years displayed some typical senescent features.^55^ Moreover, to verify the occurrence of senescence in tendons from middle-aged individuals, we also collected healthy tendons from young patients (approximately 30 years of age). All experiments were conducted in line with human research ethics and patient consent, as described in more detail in the Methods section. The histological results showed marked collagen fiber degeneration and disordered appearance in the middle-aged individuals, accompanied by a significantly higher number of P21- and γ-H2AX-positive cells (Fig. S8a–c). Similar to the findings in aged rats, human tendons from middle-aged individuals showed markedly elevated levels of p65 and pS6 expression compared to those from young individuals (Fig. S7d, e). These results demonstrate that distinct senescence occurs in tendons from middle-aged individuals at approximately 50 years old. Therefore, we isolated hTSPCs from middle-aged individuals and investigated whether POG had similar therapeutic effects on senescent hTSPCs, as found above in aged rTSPCs (Fig. 6a). Supplementation with 20 μmol·L^−1^ POG after 6 h in culture medium significantly increased the mRNA expression of SOX2 and OCT4 (Fig. 6b). In addition, POG treatment once every 2 d during a 14-d culture period significantly increased the colony formation and proliferative capacity of senescent hTSPCs (Fig. 6c). Treatment with POG also partially reduced the senescence of hTSPCs, as evidenced by a significant reduction in the number of SA-β-gal- and γ-H2AX-positive cells, as well as suppression of the senescence-related proteins P21 and P53 (Fig. 6d–g). Moreover, qRT‒PCR demonstrated that POG treatment significantly decreased the expression levels of the SASP-related genes *IL-6, MCP-1* and *IL-1β* (Fig. 6h). Intriguingly, POG treatment prior to spheroid induction also significantly enhanced the spheroid-forming capacity of senescent hTSPCs, as evidenced by the larger radius of spheroids and the higher expression of SOX2 by immunofluorescence staining (Fig. 6i, j). Notably, treatment with POG once every 2 d for 5 d prior to the onset of differentiation enhanced the tenogenic differentiation potential of aged hTSPCs (Fig. 6k). This finding was confirmed by the increased number of TNC^+^ and TNMD^+^ differentiated cells, indicating the functional rejuvenation of senescent hTSPCs (Fig. 6l).

Next, we investigated whether POG treatment could inhibit mTOR and promote autophagic activity in senescent hTSPCs. Western blotting analysis and immunofluorescence staining revealed that POG treatment suppressed the expression of pS6 and p62 and increased the expression of LC3 II/I (Fig. 6m, n). Notably, the colony-forming capacity was improved by POG treatment, and this effect was largely abolished after supplementation with 5 mmol·L^−1^ of the autophagy inhibitor 3-MA (Fig. S9a). Additionally, the application of 3-MA for 3 d counteracted the antisenescent effect of POG treatment, as evidenced by the higher number of SA-β-gal-positive cells, as well as the higher levels of γ-H2AX and p21 expression in the undifferentiated condition (Fig. S9b–d). Moreover, pretreatment with 3-MA weakened the recovery of the tenogenic differentiation potential of senescent hTSPCs with POG stimulation, as evidenced by fewer Sirius Red-positive areas (Fig. S9e). These findings further indicate that the effect of POG on senescent TSPCs relies on the simultaneous inhibition of NF-κB and mTOR and the induction of autophagy.

### Functional and structural regeneration of full-cut tendons by POG administration combined with the transplantation of biomimetic scaffolds in aged rats

**Fig. 7:**
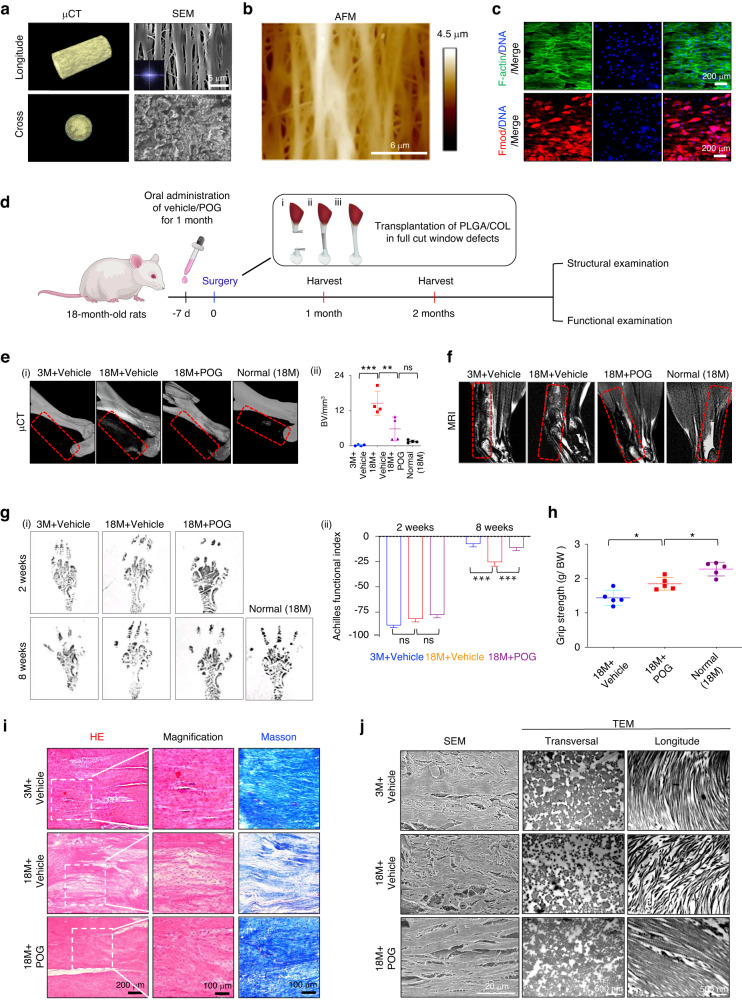
Functional and structural regeneration of full-cut tendons by POG administration combined with the transplantation of biomimetic scaffolds in aged rats. **a** μCT and SEM images of parallel-aligned PLGA-collagen scaffolds. **b** AFM height morphology of parallel-aligned PLGA-collagen fibers. **c** Schematic outlining the experimental process of the combination of POG administration. **d** Immunofluorescence staining of F-actin and Fmod. **e** (i) µCT scans of regenerated Achilles tendons. Normal (18M): Normal uninjured tendons from aged rats. (ii) Semiquantification of (i) (*n* = 5). **f** T2-weighted MRI scans of regenerated Achilles tendons. **g** (i) Footprints of experimental rats. (ii) Semiquantification of AFI (*n* = 5). **h** Grip strength of experimental rats (*n* = 5). **i** HE and Masson’s trichrome staining of neotendons (*n* = 5). **j** (i) SEM and TEM nanostructure (transverse and longitudinal) of newly formed tendon collagen fibrils. *n* = 5 rats per group. (ns: not significant; *P < 0.05; **P < 0.01; ***P < 0.001)

If a tendon is completely broken, grafts are often required to bridge.^56^ In this context, neonatal or young tendon tissues could provide favorable inductive cues, including abundant proregenerative factors and extracellular matrix, that are conducive to bridging the broken ends and activating endogenous TSPCs after a tendon injury.^21,22,57,58^ In contrast, aged tendon tissue is a notably less permissive environment for the integration of injured tissue and endogenous tendon repair.^4,8^ Above, we demonstrated that the administration of POG can rejuvenate aged TSPC functions in vitro and in vivo, which creates a relatively proregenerative environment. To further optimize the formulation of a scaffold to better bridge the broken ends for recruiting endogenous TSPCs for tendon regeneration, a hierarchical anisotropic polylactic glycolic acid/collagen (PLGA/COL) scaffold with aligned topography was fabricated as a mechanical support (~14.33 MPa) for the defect area (Fig. 7a, b, S10a). The PLGA/COL scaffold also provided a favorable environment for the growth and tenogenic differentiation of aged rTSPCs from aged rats in vitro, as evidenced by HE, Masson’s trichrome, and Sirius Red staining, as well as ordered cell spreading, as evidenced by the strong staining of the cell structure markers VINCULIN and F-ACTIN and marked increases in the expression of the key tenogenic markers TNC, TNMD, and FMOD in TSPCs after a 14-d culture period (Fig. 7c, S10b). To evaluate the in vivo biocompatibility, we implanted the PLGA/COL scaffold seeded with or without aged rTSPCs into the dorsum of athymic mice, and the transplant grafts were harvested after 8 weeks (Fig. S10c). HE staining showed that the grafts seeded with aged rTSPCs yielded dense tendon-like collagen fibers with many spindle-shaped, tenocyte-like cells. Micro- and nanostructural examination using SEM and TEM further revealed an ordered and aligned distribution of collagen fibrils, similar to that of native tendon fibrils (Fig. S10d, e). These results suggest that the PLGA/COL scaffold provides a biocompatible survival habitat for TSPC growth and differentiation.

Next, we constructed a full-cut Achilles tendon window defect with a 4 mm critical-sized gap in young and aged rats and immediately implanted PLGA/COL scaffolds only into the defect sites of rats orally administered POG or vehicle control (Fig. 7d). Without scaffold implantation, this full-cut tendon rupture with window injury would heal poorly and result in large-scale heterotopic ossification even after POG or vehicle administration (Fig. S11a, b), as reported previously.^24^ Therefore, in this experimental section, we mainly focused on the proregenerative effect of POG treatment in full-cut tendons in the context of transplantation with a biomimetic scaffold PLGA/COL (Fig. S12a). Both the aged rats orally administered POG (18M + POG) and the young rats orally administered vehicle (3M + vehicle) maintained the contours of the tendons, while the aged rats orally administered vehicle (18M+vehicle) showed a rough surface and mild hypertrophy at 8 weeks postoperatively (Fig. S12b). Microcomputed tomography (µCT) images at 8 weeks postoperatively indicated a greater degree of ectopic bone formation in the defect area in the 18M + vehicle group than in the 18M + POG group (Fig. 7e). Magnetic resonance imaging (MRI) showed a low-intensity and homogeneous signal with smooth and clear contours in the hindlimb of the 18M + POG group, comparable to the characteristics of repaired tendons in the 3M + vehicle group. In contrast, regions of high signal intensity and visible swelling were observed in the 18M + vehicle group (Fig. 7f). These results highlight the positive regulatory role of POG in inflammatory reduction.

Since tendons transfer the mechanical force created by the muscle to the bone, enabling locomotion,^1^ the locomotion performance of healing tendons by the Achilles functional index (AFI) was documented, where a more negative AFI value represents more serious hypomotility.^59,60^ At 2 weeks postoperatively, the hind paw prints of rats on the injured side in all groups were distinctly narrow, and there was no significant locomotion recovery among the groups. After 8 weeks, all AFI values increased; the 18M + POG group showed comparable values to the 3M + vehicle group and markedly higher values than the 18M + vehicle group (Fig. 7g). Considering the difference in body weight (BW) and grip strength between the young and aged rats, we modified the evaluation index in the form of grip strength/body weight (g/BW) and separately compared the value of g/BW from the tendons of the aged and young rats. The results revealed that the values in the 3M + vehicle group quickly recovered to near the level of the normal young rats (Fig. S13a). In contrast, the values in the 18M + vehicle group were significantly lower than those in the normal aged rats, whereas the values in the 18M + POG group gradually approached normal levels (Fig. 7h). To gain insights into how POG treatment rescued the endogenous tendon regenerative capacity, we performed histological analysis. Without POG treatment, sparse, disordered collagen matrix was formed in the 18M + vehicle group after 8 weeks of healing and remodeling. In contrast, the 18M + POG group achieved a more satisfactory regenerative outcome than the 18M + vehicle group, as indicated by the more compact and orderly alignment of collagen matrix deposition, which was similar to the 3M + vehicle group (Fig. 7i). This finding was coupled with the marked increases in the expression levels of the key tenogenic markers TNC and TNMD in the 18M + POG group (Fig. S13b). Similar histological results were observed in tissue sections at 4 weeks postoperatively (Fig. S13c, d). Alignment patterns and the diameter of collagen fibrils are predictors of the mechanical properties and healing of injured tendons.^61^ To confirm the relationships between these factors and neotendon formation, we evaluated the ultrastructure of the neotendon by SEM and TEM. SEM revealed that neotendons in the 18M + POG group yielded a collagen matrix with a greater density and more parallel arrangement at 8 weeks postoperatively. At the nanoscale level, more aligned and thicker collagen fibrils were observed with fibrillogenesis at 8 weeks postoperatively in the 18M + POG group (Fig. 7j). Mechanistically, POG administration significantly reduced the number of P21^+^, γ-H2AX^+^, and pS6^+^ cells in the process of tendon repair. Furthermore, POG administration significantly reduced the expression of IL-6 in neotendons in the 18M + POG group compared to the 3M + vehicle and 18M + vehicle groups (Fig. S14a–c).

## Discussion

As the aging population grows worldwide, the development of therapies to rejuvenate deteriorated physiological functions and ameliorate aging-related symptoms has become an important topic in the scientific field.^62,63^ Like most tissue regenerative processes, the regenerative capacity of tendons decreases with aging or even fails and is often accompanied by stem cell exhaustion and cellular senescence.^4,9^ To improve the regenerative potential of aged tendons, in this study, we utilized a newly developed system, namely, DLEPS, to identify small molecules from natural compounds based on a Yamanaka factor score and the gene expression profiles from different tendon development stages. The in vitro serial passage-induced cellular senescence model has been proven to be an accessible and straightforward cell senescence model for tentatively estimating the antisenescent effect of several key genes and compounds, despite some differences from the in vivo natural aging model, including metabolic factors or inflammation.^26–28,64^ In our study, we found that the top-ranked POG could efficiently inhibit TSPC senescence and promote their tenogenic differentiation potential in an in vitro serial passaging cell senescence model. We also found that the top-ranked POG potently rejuvenated the proliferation and tenogenic potential of TSPCs from both aged rats and middle-aged humans by maintaining stemness and suppressing senescence. Due to the risk of sample surgery in individuals who are too old (ages above 70 years) and difficulty in communicating with their family members, we isolated senescent TSPCs from donors who were approximately 50 years old and displayed senescent features that were similar to those in a previous study.^55^ In the future, we expect to obtain senescent TSPCs from older individuals and examine whether POG is also able to restore more serious hypofunctional conditions. Generally, the results from multiple senescent cell models provide solid and convincing evidence that POG is indeed a potent antisenescent drug for TSPCs. Moreover, the systemic administration of POG and the local delivery of POG-nps were found to promote aged tendon self-repair in small-sized, partial transection tendon injuries. The combination of POG administration and the transplantation of PLGA/COL scaffolds significantly enhanced the aged endogenous regenerative capacity in large-sized, full-cut tendon window defects in aged rats. These findings provide multiple alternative strategies for endogenous tendon repair and regeneration in aging according to different injury conditions.

The regenerative capacity of adult tendons depends on the status of TSPCs, which can activate and expand to form new tendon collagen fibers or self-renew to restore the TSPC pool in response to tissue damage.^7^ At later stages in life, TSPCs present a marked impairment of stemness and regenerative capacity, resulting in inefficient tendon self-repair.^4,8^ In previous research, we found that the difference in TSPC stemness between the neonatal and adult stages influenced multiple biological functions of TSPCs and the adult tendon regenerative process.^22^ In this context, a reasonable stemness-modulating method is a prospective strategy for remedying aged TSPCs. Traditionally, the dampened stemness can only be reversed by inducing Yamanaka factors genetically.^13,14^ However, an efficient strategy to identify stemness-promoting small molecules is currently lacking. In recent years, deep learning approaches have been widely applied in target-based drug design and for the development of various therapeutic strategies.^65,66^ However, these methods cannot be used when the target is unknown; for instance, with regard to stemness promotion, only four transcription factors are known. In this study, we employed the newly developed system, DLEPS, which is an efficacy prediction system using transcriptional profiles with deep learning,^23^ to identify potential drugs to stimulate stemness. Based on DEGs in the regeneration of neonatal and postnatal tendons,^22^ we found that POG, which is inaccessible using target-based methods, significantly rescued aging-impaired TSPC functions and tendon regenerative capacity. Our findings are consistent with the performance of DLEPS in efficiently predicting molecules with desired transcriptional changes in obesity and hyperuricemia.^23^

It has been demonstrated that NF-κB can cause stem cells to become senescent and aggravate cell hypofunction.^41,67^ IL-6 and MMP9 act as inflammatory aging-related genes that are regulated by NF-κB, which is also an indicator of the activation of the pathway.^68–71^ In accordance with this, our results from in vivo experiments also showed that POG treatment could inhibit the expression of IL-6 and MMP9. Since TNF is widely used as an inducer to simulate the activation of the NF-κB pathway in mesenchymal stem cells in vitro,^41,72,73^ we also demonstrated that POG was able to inhibit the TNF-induced activation of the NF-κB pathway in TSPCs in vitro by Western blotting and immunofluorescence. Generally, we demonstrated that POG could inhibit the overactivation of NF-κB and SASP expression. Interestingly, existing studies have found that POG treatment can inhibit the activation of NF-κB in Raw264.7 cells,^29^ which was also consistent with our findings to some extent. The inhibition of a single signaling pathway or cellular process could partially counteract several hallmarks of senescence. However, similar to various existing antisenescent drugs, it is unlikely that an effective compound has rejuvenescent and antisenescent functions, widely considered a complex process, on the basis of a single mechanism.^74^ The mTOR pathway is often overactivated during stem cell senescence and aged tissue regeneration.^32^ Notably, the transient activation of autophagy via the Sox2-mediated suppression of mTOR is a critical step in reprogramming to pluripotency.^46^ By using both in vitro cellular senescence and in vivo aging models, we found that as senescence progressed, the expression of pS6, an important downstream factor of the mTOR pathway, markedly increased in TSPCs. Existing applications in other tissues have not involved the senescence background, which may explain why reports on the modulatory effect of POG on the mTOR pathway are lacking. Autophagy is a conserved lysosomal degradation pathway essential for cellular homeostasis and is impaired in age-related diseases. Many physical and pharmacological interventions that extend lifespans, such as caloric restriction and rapamycin, can induce autophagy.^48,49^ Moreover, the induction of autophagy is regarded as a decisive factor between the stemness and senescence fate of stem cells.^47^ Autophagic activation is often accompanied by the inactivation of the mTOR pathway.^46,47^ In this study, we found that POG was able to enhance autophagic activity in senescent TSPCs in serial-passaging and natural-aging cell models. More importantly, we found that when the E1-like activating enzyme ATG7 was knocked down, the beneficial effect of POG was completely counteracted. However, the direct drug target of POG was not determined in this study. Identifying the direct target would be beneficial to optimize POG and minimize potential side effects. Additionally, elucidating new drug targets could guide disease diagnosis efforts. In the future, we still need to discover direct protein targets in TSPCs by which POG inhibits the activation of the mTOR and NF-κB signaling pathways. Overall, we found that POG exerts a double-node inhibition of both the mTOR and NF-κB signaling pathways, which suggests that dual inhibition of both mTOR and NF-κB is a highly efficient combination for all double-node inhibition stimulations. It has been reported that immune cells also play vital roles in tendon aging or repair.^75,76^ Macrophages have been demonstrated to be key modulators of tendon repair and regeneration.^77^ As major participants in the early stage of tendon repair, macrophages can communicate with tendon-resident cells through paracrine signaling to regulate their function.^35^ Although we demonstrated that POG could inhibit the expression of inflammatory factors in vivo to some extent, we may need to explore whether POG could influence specific immune cells, including macrophages and neutrophils, which eventually modulate the progression of tendon aging or repair.

Older individuals and patients with tendon rupture often have a weakened reparative capacity due to the dysregulation of TSPCs in their microenvironment.^4,9^ To overcome this, we developed POG-nps for use as a local therapeutic intervention for the repair of small-sized and partial-transection tendon injuries. Notably, POG-nps could be conveniently injected into injured tendons in aged rats, whereby the controlled release of POG effectively modulated the local number of TSPCs and their tenogenic potential to promote the repair of damaged tendons (Fig. 3). Previous studies have reported that lipid droplet deposition can hamper tendon regeneration.^78^ We found that POG-nps exerted additional therapeutic effects by removing lipid droplets, possibly due to the impact of mTOR inhibition by POG. Compared to small-sized, partial-transection tendon injuries, large-ranged, full-cut window tendon defects often result in poor healing and the development of heterotopic ossification.^22^ Therefore, we fabricated a hierarchical anisotropic PLGA/COL scaffold with aligned topography, which provided a favorable environment for TSPC growth and tenogenic differentiation. However, poor retenogenesis during tendon aging hampers biomaterial-mediated tendon regeneration with impaired TSPC functions, as well as a delay in the differentiation of TSPCs into tenocytes.^4,8^ Therefore, matching measures to assist in biomaterial-mediated tendon regeneration are important. By combining the PLGA/COL scaffold with the systematic use of POG, we were able to restore structural and functional tendon regeneration in aged rats by inhibiting cellular senescence and significantly reducing the inflammatory response. The transformation can be realized, in principle, by providing a more youthful systemic environment that is conducive to tendon regeneration, which promotes the intrinsic functions of TSPCs. Although we obtained satisfactory antisenescence and regenerative results in 18-month-old aged rat models without systemic toxicity (Fig. S15), older rats at approximately 24 months old may need to be utilized to examine whether POG is able to reverse the hampered regenerative capacity that was induced by intensified senescent conditions. Furthermore, existing studies have conducted preliminary examinations on the pharmacokinetics of orally administered POG.^79,80^ These studies found that POG is primarily transformed into cimifugin upon absorption into the bloodstream. This transformation prompted us to further explore the biological importance of the mechanism.

**Fig. 8:**
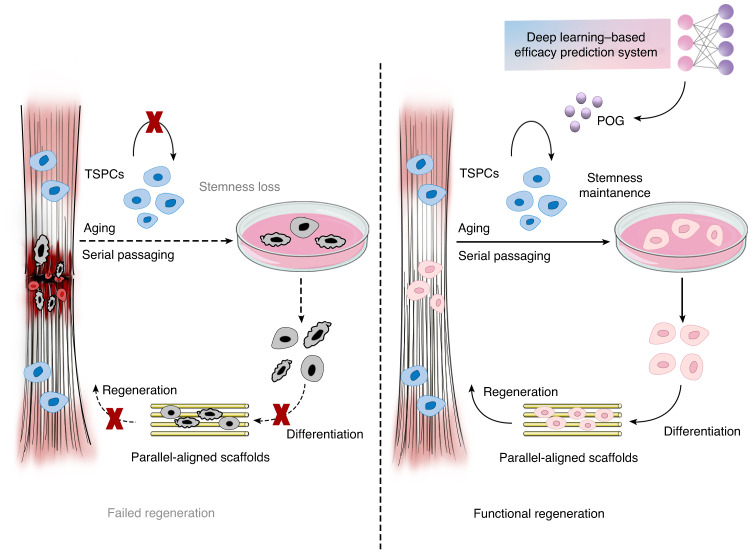
Schematic diagram highlighting that deep learning-predicted prim-O-glucosylcimifugin (POG) rescues aging-impaired tendon stem/progenitor cell (TSPC) stemness and functions by dual targeted inhibition of mTOR and nuclear factor-κB. The systemic administration or local delivery of POG functionally promotes endogenous tendon repair and regenerative capacity in aged rats

In summary, this study employed a DLEPS to uncover a potential stemness-promoting drug, POG, which rescues aging-impaired TSPC stemness and functions by dual targeted inhibition of mTOR and nuclear factor-κB and activation of autophagy. The systemic administration of POG or the local delivery of POG nanoparticles restored structural and functional tendon regeneration in aged rats without transplanted cells (Fig. 8). These findings possess great translational potential for clinical tendon therapies.

## Materials and Methods

### Animals

All the experimental procedures in this study were conducted in line with animal welfare ethical regulations and approved by the Animal Use and Care Committee of Peking University (LA2018302). Male 3-month-old (young rats) and 8-month-old (8M) Sprague‒Dawley rats and 6–8-week-old BLAB/c nude mice were purchased from Wei Tong Li Hua Experimental Animal Center (China). The 8M rats were further housed for ten months to be used for 18M aged rat experiments. All animals were housed in ventilated cages on a 12:12-h light/dark cycle with ad libitum access to food and water. Rats and mice were removed during different experiments if they died prematurely or suffered from tumors.

### Oral administration of POG

Generally, aged rats were fed ad libitum and administered POG (Topscience, Cat# 62499-28-9) in their drinking water (50 mg·kg^−1^ BW per day). In the experiments examining uninjured tendons from aged rats, they were also fed drinking water (50 mg·kg^−1^ BW per day). All animals were housed in pairs or groups of up to four animals. Fluid consumption was continuously monitored to adapt dosages. All animals were randomly assigned to experimental groups.

### Partial transection of Achilles tendons

In this section, oral administration of POG started at two months prior to the surgery in rats. POG treatment continued until one day before the partial transection. For partial transection, the rats were injected intraperitoneally with 1% pentobarbital (0.1 mL per 100 g), and then, the rats were completely anesthetized. The Achilles tendons were exposed through a lateral transection under general anesthesia. A gap wound model (half in width) of the Achilles tendons was performed using micro-operating instruments for each leg. The wound was then irrigated, and the skin was closed with a suture. Postoperatively, rats were allowed free cage activity at a constant temperature with a 12-h dark-light cycle and unrestricted access to a standard diet and water. In consideration of the old age of the animal subjects, we selected unilateral surgeries to reduce the surgical pain of rats. For the partial-transection experiments in Figs. 2 and 3, we selected 1 and 4 weeks post-injury as the time points when the rats were sacrificed for histological evaluation at each time point. Moreover, 5-bromo-2-deoxyuridine (BrdU, Sigma) at a concentration of 80 mg·kg^−1^ was intraperitoneally injected daily starting the day after partial transection in each group.

### Isolation of TSPCs

Primary TSPCs from rats and humans were isolated from Achilles tendons according to the previously established procedure, in which the selected TSPCs met the criteria of mesenchymal stem cells.^22^ Briefly, the harvested Achilles tendons were minced and digested fully in 3 mg·mL^−1^ type I collagenase (Thermo Fisher Scientific, Cat# 17100017) and 4 mg·mL^−1^ dispase (Roche, Cat# 10269638001) at 37 °C for 0.5 h. After filtration through a 70 μm strainer, single-cell suspensions were cultured in low-glucose Dulbecco’s modified Eagle’s medium (DMEM, HyClone, Cat# SH30021.01B) with 15% fetal bovine serum (FBS, Thermo Fisher Scientific), 2 mmol·L^−1^ glutamine (Thermo Fisher Scientific, Cat# 25030081), and 100 U·mL^−1^ penicillin/streptomycin (Thermo Fisher Scientific, Cat# 15070063). Upon reaching 80%–90% confluence, cells were trypsinized, centrifuged, resuspended in growth medium as passage two cells, and incubated in 5% CO_2_ at 37 °C, with fresh medium changing every 2–3 d. Most cultured cells displayed fibroblast-like morphology after 2 or 3 passages and then were trypsinized, resuspended, and seeded onto 96-well plates at low density (2–4 cells per cm^2^) with 100 μL of medium. After 7–10 d, colonies were examined by microscopy and labeled carefully. Then, the plates were gently detached carefully by trypsin-EDTA solution (HyClone, Cat# SH30042.01). Selected colonies were subcultured into a 12-well plate.

### Flow cytometry

We incubated 1 × 10^6^ rat TSPCs with primary antibodies against CD90, CD105, CD29, CD45, and CD34 for 1 h and then incubated them with fluorescent secondary antibodies for 1 h at 4 °C. Finally, we analyzed the samples using a flow cytometer (BD Accuri C6) to calculate the expression of cell surface markers in isolated TSPCs (Fig. S1).

### Cell proliferation assay

For rTSPCs in serial passaging experiments, 1 × 10^4^ cells were seeded on a 24-well plate for 48 h. For POG- or DMSO-treated aged rTSPCs from aged rats or hTSPCs, 1 × 10^4^ cells were stimulated with 20 μmol·L^−1^ POG or DMSO once every 2 d for 5 d. All cells were then fixed and permeabilized for Ki67 immunofluorescence.

### Three-dimensional spheroid formation assay

For rTSPCs in serial passaging experiments, the cell suspension harvested from the adherent monolayer of rTSPCs was stopped with stimulation by POG. Cells were then plated in Corning Costar Ultra-Low Attachment 6-well plates in serum-reduced DMEM with 10 ng·mL^−1^ FGF2 (Peprotech, Cat# 400–29) and 20 ng·mL^−1^ EGF (Peprotech, Cat# 400–25). Collected spheroids were observed and counted under the microscope on Days 7–10. Then, the spheroids were transferred into normal attachment 6-well or 12-well plates (Corning, Cat# 3474) with Matrigel (Corning, Cat# 354234) and cultured with tenogenic medium. Immunofluorescence staining was used to analyze the tenogenic differentiation capacity. SEM was used to examine the microstructure of newly formed collagen fibers.

For POG- or DMSO-treated aged rTSPCs from aged rats, cells were seeded onto 12-well plates and stimulated with 20 μmol·L^−1^ POG or DMSO once every two days for 5 d. Then, cells were induced into cell spheroids and examined by immunofluorescence.

### Computation of enrichment score

Previously, we described a deep-learning-based efficacy system (DLEPS) in *Nature Biotechnology*.^23^ DLEPS is the first efficacy prediction system as far as we know that employed variational autoencoder to encode the small molecules in Simplified Molecular Input Line Entry System (SMILES) format and predicted the changes of transcriptional profiles (CTPs) using the dense network from latent vectors. As we have shown, this method was able to predict the CTPs at very high accuracy (the mean correlation coefficient of CTPs is 0.74, while the peak value is 0.92). After prediction of CTPs for 12 328 genes, the Kolmogorov‒Smirnov (KS) test was used to evaluate the distribution of query genes in the list of ranked genes. The enrichment score for the up (*a*)/down (*b*) gene set is defined as follows:

$$a=\mathop{\max }\limits_{j=1\,to\,t}\left[\frac{j}{t}-\frac{V(j)}{n}\right]$$

$$b=-\mathop{\max }\limits_{j=1\,to\,t}\left[\frac{V(j)-1}{n}-\frac{j-1}{t}\right]$$

where *t* is the number of genes in the query gene set, *n* is the number of genes in the computed CTPs, and *V(j)* is the rank of a specific gene in the rank list. The enrichment score is defined as follows:

$${\rm{Score}}=\left\{\begin{array}{ll}a-b & when\,b\ast a \,<\, 0\\ 0 & when\,b\ast a \,>\, 0 \end{array}\right.$$

Note that (–1) × [*a −* *b*] or (*b* – *a*) means to reverse the gene signatures. The microarray datasets have been submitted to the NCBI database under the accession number GSE158342.

### Small molecule libraries

In this study, the compound list remained the same as that in the previous study.^23^ The Natural Compound Library (L6000, TargetMol, US, *n* = 2 719) and FDA-approved library (L4200, TargetMol, US, *n* = 961), merged as D3680, were used to screen the positive chemicals modulating TSPC stemness.

### Identification of gene signatures

We used both customized RNA-seq data and the known Yamanaka factors to calculate the tendon score. Generally, the Yamanaka factor score reflects the extent to which a small molecule can promote the stemness of rTSPCs. The tendon score was calculated using the DEGs between 6–8 weeks and 0–1 d rat tendon samples. The DEGs were ranked by fold change and filtered by *P* value with a threshold of 0.05. Finally, we obtained 99 upregulated and 143 downregulated genes, which were used as input for DLEPS.

We intended to use five stemness factors, Nestin, Sox2, DDX56, OCT4 and Nanog, while DDX56 and OCT4 were not in the 12 328 gene list. We thus used the other three genes; two of them were very well predicted, as shown in Fig. 1b. The Pearson correlation coefficients of Nestin, Nanog and Sox2 were 0.77, 0.78 and 0.51, respectively. Finally, we filtered the compounds using both stemness and tendon scores, as shown in Fig. 1c and Table S1.

### LysoTracker staining

rTSPCs from young and aged rats without or with POG stimulation were incubated in prewarmed medium containing 1:5 000 LysoTracker RED (Beyotime, CAT#L7528) for 30 min with DAPI at 37 °C. Cells were washed twice with fresh media before imaging. For analysis of LysoTracker staining under different conditions, the region of interest around each cell was drawn for measurement and analysis by a blinded observer using ImageJ. At least 50 cells per replicate were analyzed. Each LysoTracker experiment was repeated at least three times.

### In vivo subcutaneous transplantation

For rTSPCs in serial passaging experiments, 1 × 10^6^ rTSPCs at P12 with or without POG treatment were cultured on the collagen sponge for 24 h. For DMSO-treated, POG-treated and ATG7 knockdown aged rTSPCs from 18-month-old rats, 1 × 10^6^ cells were stimulated with 20 μmol·L^−1^ POG once every two days for 5 d and then transferred to the collagen sponge. Then, a collagen sponge was implanted subcutaneously in the dorsum of athymic mice (6–8 weeks, BALB/c nude mice) under anesthesia with isoflurane. After confirmation of blood flow and hemostasis following removal of the clamp, the wound area was closed. Finally, implants after 8 weeks of retention were retrieved and then fixed with 4% paraformaldehyde. Paraffin sections were harvested at a thickness of 4 μm, followed by HE and immunofluorescence staining. For comparison the effect with or without seeded TSPCs in nude mice, 18 mice with 9 mice in each group were used.

### Preparation of drug-loaded PLGA nanoparticles and PLGA/COL scaffolds

Drug-loaded PLGA nanoparticles were prepared using an emulsification solvent evaporation technique. In brief, PLGA/PLGA-b-PEG (50/50, w/w; Ruixi) was dissolved in dichloromethane to make 10% w/v solutions. Then, 5% (w/w) of chemicals (POG with the same molar ratio) to the polymer weight were codissolved in the polymer solution. The resulting solution was added to a stirred 1% (w/v) PVA solution using a vortex mixer at 2 000 r·min^−1^ for 2 min and sonicated with an amplitude of 20% for 60 s. After sonication, the emulsion was added dropwise into 1% PVA and stirred for 3 h at room temperature to remove the residual organic solvent. The nanoparticles were collected and washed three times with distilled water by centrifugation at 10 000 × *g* for 5 min at 4 °C.

A biomimetic parallel-aligned tendon scaffold was fabricated by electrospinning at a voltage of 15 kV. Then, 20% (w/v) PLGA (lactide/glycolide = 60:40, Mw = 105 kD, Sigma-Aldrich, Cat#900300) and 2% (w/v) type I collagen (Corning, Cat#54236) solutions were dissolved in 1,1,1,3,3,3-hexafluoro-2-propanol solvent (Sigma-Aldrich, Cat#920661) and added into a plastic syringe equipped with a flat-tipped 21-gauge (G) needle. The distance was set as 15 cm between the needle and the collector. The rotational speed of the collecting drum was set at 2 400 r·min^−1^. For crosslinking of collagen, the membranes were immersed in an 80% ethanol solution containing 5 mmol·L^−1^ 1-ethyl3-(3-dimethylaminopropyl) carbodiimide hydrochloride (Sigma-Aldrich, Cat# 25952-53-8) for 24 h at 4 °C. Then, membranes were cryopreserved at −80 °C for 2 h and dried in a vacuum oven for 24 h. Subsequently, the prepared membranes were sterilized using γ-irradiation for in vitro and in vivo experiments.

### In vitro experiments with POG-loaded nanoparticles

Aged rTSPCs from aged rats were seeded in the corresponding plates according to different experiments. Generally, POG-loaded PLGA nanoparticles (20 μmol·L^−1^, POG-nps) diluted in PBS were added to the plates. Considering the drug-releasing characteristics of POG-nps, we only added them once and then changed half of the POG-nps every other day. For Ki67, P53 and P21 examinations, we stimulated aged rTSPCs with POG-nps for 48 h. For tenogenic induction, we first incubated the aged rTSPCs in the POG-nps for 4 d. Then, we transformed the undifferentiated medium into tenogenic medium to ensure that POG-nps were removed during tenogenic induction.

### Drug release assay

POG-nps (2 mg) were dispersed in 0.22 μm filters inserted in a centrifuge tube (Corning) with continuous shaking at 37 °C. At the indicated time points in Fig. 3, 3 mL of solution was obtained and frozen for subsequent examinations. Aliquots of sample were measured by HPLC–MS (Agilent 6460).

### Atomic force microscopy

The nanostructure of the PLGA/COL scaffold was tested using an atomic force microscope (AFM, Dimension Icon, Bruker, USA) operated under the peak-force tapping mode with a 1.0 Hz scan rate and a 250 mV amplitude set point. A TAP150A probe was used, and deflection sensitivity was calibrated on the sapphire model. Data were analyzed using Nanoscope Analysis software 1.60.

### Mechanical test of scaffolds

The mechanical properties of the as-fabricated PLGA/COL scaffold were analyzed using tensile testing equipment (3345, Instron, USA). Briefly, measurements of the cross-sectional area of scaffolds were performed using alginate dental impression paste. The scaffold was then fixed to custom-made clamps using high-strength thread. After application of a preload of 0.1 N, each complex was cyclically elongated between 0 and 0.5 mm for 20 cycles at 5 mm·min^−1^ in advance. Subsequently, the complex underwent a load-to-failure test at a 5 mm·min^−1^ elongation rate. The load–elongation behavior of the complex and failure modes was recorded. The mechanical properties of the scaffold were calculated by failure force (N) and modulus (MPa).

### Full-cut window tendon defects

In this section, oral administration of POG started at 7 d prior to the surgery in both groups of rats. POG treatment was interrupted for 3 d before and 3 d after surgery and then commenced to 4 weeks after surgery. The rats were injected intraperitoneally with 1% pentobarbital (0.1 mL per 100 g) to anesthetize them. For a full-cut Achilles tendon defect, the central one-third of the Achilles tendon (~4 mm in width for rats) was removed from the distal apex of the muscle to the insertion of the calcaneus. Since the complete defect area showed an obvious shrinkage without scaffold implantation,^24^ the scaffold was inserted in the tendon defect for all the groups and sutured to the broken ends using Ethicon 6–0 suture. After the operation, the operated limbs were immobilized for 1 weeks. In consideration of the old age or pathological condition of the animal subjects, we selected unilateral surgeries to reduce the surgical pain of rats. Notably, we selected 2 weeks and 8 weeks as the time points to examine the AFI index in living bodies. To examine the histological appearance, we selected 4 weeks and 8 weeks as the time points when rats were sacrificed for histological evaluation at each time point.^22^

### In situ tendon regeneration with POG-loaded nanoparticles

The aged rats were injected intraperitoneally with 1% pentobarbital (0.1 mL per 100 g) to anesthetize them, and a partial transection injury was created for in situ regeneration as described above. POG-loaded PLGA nanoparticles (80 μmol·L^−1^, POG-nps) suspended in 50 μL of PBS were injected into injured tendons. As the control, the contralateral tendons of recipient rats were similarly injured but injected with PLGA nanoparticles without POG. Five rats per group were used for each time point. The animals were given food and water ad libitum.

For visualization of the distribution of nanoparticles in the injured tendon after injection and confirm the retention of nanoparticles, Cy5 was added in the synthetic process of PLGA to obtain PLGA-Cy5. Rats were injected with PLGA-Cy5, and the in vivo nanoparticle distribution was analyzed with a fluorescence imaging system at the indicated times. Considering the old age of the animal subjects, we selected unilateral surgery to reduce the surgical pain of the animals.

### Macroscopic evaluation of regenerated tendons

At 8 weeks postoperatively, rats were euthanized, and the Achilles tendons were exposed fully and photographed using a camera (Nikon, Japan). Then, the full-length tendon complexes, including partial gastrocnemius and calcaneus, were harvested.

### Examination of the Achilles functional index

For evaluation of the healing outcomes of the Achilles tendons, their motility was assessed by the AFI at setup time points in different in vivo experiments, adopting a modified method from a previous study.^56^ Briefly, a restrictive roadway (100 cm long and 25 cm wide) was covered with white paper. After their hind paws were evenly dipped with black ink, the rats were allowed to walk freely, and black footprints were printed on white paper. For quantification of the AFI of the rats, footprints were scanned (Epson Scanner, Japan). We defined and acquired related footprint parameters, including print length (PL), toe spreading length (defined as the distance between the first and fifth toes, TS), and intermediary toe spreading length (defined as the distance between the second and fourth toes, IT). Then, according to the difference between the normal (N) and the experimental values (E), three footprint dimension factors (PLF, TSF, ITF) could be acquired using the following equations:

PLF = (NPL−EPL)/EPL (1) TSF = (ETS−NTS)/NTS (2) ITF = (EIT−NIT)/NIT (3) Finally, the AFI was calculated according to an established equation^56^

$${\rm{AFI}}=74({\rm{PLF}})+161({\rm{TSF}})+48({\rm{ITF}})-5$$

### Strength by grip test

Rats were allowed to grasp a horizontal grid connected to a dynamometer (Sansbio, CAT#SA417) with a hindlimb and were pulled backward five times. The force applied to the grid each time before the animal lost its grip was recorded in Newtons. To avoid the effect of individual variation to the greatest extent, we sequentially performed five tests. The results are expressed as the mean of 3 median values in grams (g) and normalized by body weight (g/BW).

### Magnetic resonance imaging

Rat Achilles tendons were subjected to MRI scans using an 8-channel surface coil at 3.0 T (Siemens, Germany) at 8 weeks postoperatively for five rats from each group. Acquisition parameters were as follows: sagittal T2-weighted imaging (Turbo spin echo, repetition time = 3 104 ms, echo time = 80 ms, field of view = 90 × 110 mm, matrix size = 384 × 384, slice thickness = 1 mm, interslice distance = 0.1 mm).

### Microcomputed tomography

Hindlimbs from rats were fixed in 4% paraformaldehyde and analyzed by µCT (Skyscan 1172). The scanner was set at a voltage of 80 kV and a resolution of 18 mm per pixel. The images were analyzed by NRecon, CTAn, and CTVol software.

### Scanning electron microscope

The microstructures of scaffolds, nanoparticles, neotissues and cell membranes after tenogenic induction in Fig. S3f were examined using SEM (Hitachi SU8020, Japan) at 10 kV. Cell membranes and neotissues were prefixed in 2.5% glutaraldehyde in PBS (pH 7.4) at 4 °C for 12 h and washed three times with PBS. All the samples were dehydrated in a graded series of ethanol (50%–100%), critical-point dried, and sputter-coated with gold for 2 min at 20 mA.

### Transmission electron microscopy

Nanoparticles and neotissues were examined by TEM (Hitachi HT7700, Japan) at 100 kV. For neotendons, samples were double-fixed with 1% osmium tetroxide (Polysciences, Cat#23311-10), stained with lead citrate and uranyl acetate, and embedded in epoxy resin. Transverse and longitudinal ultrathin sections of 70–100 nm were cut with a Leica ultramicrotome and collected on copper grids.

### Statistical analysis

Statistical analysis was performed using GraphPad Prism software 8 (GraphPad Software, Inc.). All the experimental results are expressed as the mean ± SD. Differences between groups were assessed by unpaired two-tailed *t* tests (for two-sample comparisons) or one-way analysis of variance with Tukey’s post hoc test (for multiple comparisons). Statistical significance was set at *P* < 0.05.

### Supplementary information

Revised Supplementary Materials

## Data Availability

The accession number for the RNA-seq datasets reported in this paper will be uploaded when requested. Any other requests for raw or processed data will be reviewed by Peking University National Engineering Laboratory for Digital and Material Technology of Stomatology to verify whether the data requested are subject to any intellectual property or confidentiality obligations. The microarray data are deposited at NCBI Gene Expression Omnibus “GSE241201” and “GSE241202”.
