# Supplementary material for: Prim-O-glucosylcimifugin ameliorates aging-impaired endogenous tendon regeneration by rejuvenating senescent tendon stem/progenitor cells
Source: Bone Res. 2023 Oct 23;11:54. doi: 10.1038/s41413-023-00288-3 (PMC10593834; doi:10.1038/s41413-023-00288-3)
Supplement: Supplementary file 1 — Revised Supplementary Materials [file 41413_2023_288_MOESM1_ESM.docx]

Supplementary Materials for

**Prim-O-glucosylcimifugin ameliorates aging-impaired endogenous tendon regeneration capacity via rejuvenating senescent tendon stem/progenitor cells**

Yu Wang^1^, Shanshan Jin^1^, Dan Luo^2^, Danqing He^1^, Min Yu^1^, Lisha Zhu^1^, Zixin Li^1^, Liyuan Chen^1^, Chengye Ding^1^, Xiaolan Wu^1^, Tianhao Wu^1^, Weiran Huang^3^, Xuelin Zhao^4^, Meng Xu^4^, Zhengwei Xie^3,5*^, Yan Liu^1,5*^

^1^Laboratory of Biomimetic Nanomaterials, Department of Orthodontics, Peking University School and Hospital of Stomatology & National Center for Stomatology & National Clinical Research Center for Oral Diseases & National Engineering Laboratory for Digital and Material Technology of Stomatology & Beijing Key Laboratory of Digital Stomatology & Research Center of Engineering and Technology for Computerized Dentistry Ministry of Health & NMPA Key Laboratory for Dental Materials & Translational Research Center for Orocraniofacial Stem Cells and Systemic Health, Beijing 100081, China

^2^CAS Center for Excellence in Nanoscience, Beijing Key Laboratory of Micro-nano Energy and Sensor, Beijing Institute of Nanoenergy and Nanosystems, Chinese Academy of Sciences, Beijing 101400, China

^3^Peking University International Cancer Institute, Health Science Center, Peking University, Beijing 100083, China

^4^Department of Orthopedics, the Fourth Medical Center of PLA General Hospital, Beijing 100048, China

^5^These authors jointly supervised this work: Yan Liu, Zhengwei Xie.

^*^Email: [orthoyan@bjmu.edu.cn](mailto:orthoyan@bjmu.edu.cn); [xiezhengwei@hsc.pku.edu.cn](mailto:xiezhengwei@hsc.pku.edu.cn).

**Supplemental Materials and Methods**

**Human tendon samples:** The human tendon samples were harvested from the collaborative team in the General Hospital of the People’s Liberation Army. The use of them was in compliance with ethical regulations from clinical trials medical ethics committee, the 4th medical center of General Hospital of the People’s Liberation Army (PLAGH) (2021KY045-KS001). According to the objective of this study, we established strict inclusion criteria to obtain suitable human samples. In thought of difficulty in accessing human tendons in our clinic, all patients were recruited from the General Hospital of the People’s Liberation Army where the structural integrity of tendons was determined ultrasonographically. Because it was extremely difficult to obtain completely healthy tendons or get consent from healthy individuals, we set the patients (around 30 years old) who required tendon excision due to severe trauma as young groups; the patients (around 50 years old) were set as relatively aged groups, in the view of the risk in sample surgery in too old individuals (ages above 70 years) and difficulty in communication with their family members. The patients who have severe systemic inflammatory diseases and rheumatoid arthritis were excluded to avoid influence of non-aging factors. All the patients lived in Peking or northern areas of China. At least 10 individuals were collected in two groups. A small part of normal tendon tissues was harvested from the regions away from the surgical margins to avoid the influence of trauma. To further verify the anti-senescent function of POG, we selected the hTSPCs from the relatively aged group as the study object. We found γ-H2AX and P21 positive cells in harvested tissues (Fig. S8b, c), which could confirm the reasonability of sample usage.

***In vitro* serial passaging experiments:** rTSPCs from young rats were passaged from P3 to P12. POG was dissolved in DMSO solutions. In order to exclude the cytotoxicity of DMSO, we keep 20mM as working solutions. During serial passaging, POG solutions were added to the culture medium at concentration of 20 μM in each passage, and an equal amount of DMSO that reach 0.1% volume ratio was added in the control groups to exclude the influence of solvent **(**Fig. 1f**)**. When rTSPCs were passaged until the 12th passage, we stopped the supplementation of POG in the culture medium and performed subsequent examinations.

**CFU-F assay:** For rTSPCs in serial passaging experiments, 2x10^3^ cells were seeded onto 6-well plates and cultured in the growth medium for 14 days. For POG- or DMSO-treated aged rTSPCs from aged rats or hTSPCs, 2x10^3^ cells were seeded onto 6-well plates and stimulated with 20 μM POG or DMSO once every 2 d for 7 d. CFU-F colonies were stained with 0.5% crystal violet (Solarbio, Cat#G1062) in 4% Paraformaldehyde and counted.

**SA-β-gal assay**: For rTSPCs in serial passaging experiments, cells were seeded onto 12-well plates for 48 h. For POG- or DMSO-treated aged rTSPCs from aged rats or hTSPCs, cells were seeded onto 12-well plates and stimulated with 20 μM POG or DMSO once every 2 d for 5 d. For human tendon samples, the tissues were resected and fixed in 4% paraformaldehyde, decalcifified with 10% EDTA (pH 7.0) for 7 d and embedded in optimal cutting temperature compound. Then, 6-μm thick longitudinal sections were lay on a 24-well plate for 24 h. Tissue sections and cells were analyzed using the SA-β-gal staining kit (Cell Signaling Technology, Cat#9860) according to the manufacturer’s instructions.

**Tenogenic induction assay:** For rTSPCs in serial passaging experiments, cells were seeded onto 12-well plates. Cells were stopped with stimulation by POG and cultured in tenogenic differentiation medium containing 10 ng/mL TGF-β1 (Peprotech, Cat#AF-100-21C), 10 ng/mL GDF-5 (R&D Systems, Cat#853-G5), 0.05 mM l-ascorbic acid 2-phosphate (Sigma-Aldrich, Cat#A5960) that have been demonstrated to be able to induce tenogenic differentiation **^[10]^**. For POG- or DMSO-treated rTSPCs from aged rats or hTSPCs, 2x10^3^ cells were seeded onto 12-well plates and stimulated with 20 μM POG or DMSO once every 2 d for 5–7 d. Then cells were cultured in differentiation medium for another 7 d without POG addition. For Sirius Red and Masson’s trichrome staining, cells were fixed with 70% ethanol for 1 h and stained according to the corresponding staining kit (Abcam, Cat#AB150681; Solarbio, Cat#G1340) respectively.

**qRT-PCR and RNA-seq:** For qRT-PCR, total RNA was extracted using Trizol reagents (Thermo Fisher Scientiﬁc, Cat#15596026) according to the manufacturer’s instructions. Then RNAs were transcribed to complementary DNA by reverse transcriptases. qPCR was performed using gene-speciﬁc primers and SYBR Green (Thermo Fisher Scientiﬁc, Cat#4385612) on 7900HT Fast Time PCR. The primer sequences synthesized were listed in Supplementary Table 4.

For RNA-seq, rTSPCs at P12 and aged rTSPCs from aged rats treated with DMSO or POG were harvested. Total RNAs were isolated using Trizol reagents (Thermo Fisher Scientiﬁc, Cat#4385612) and puriﬁed with a RNeasy mini kit (Qiagen, Cat#74104). Biotinylated cDNA was prepared according to the standard Affymetrix protocol from 150 ng total RNA by using Ambion® WT Expression Kit. Following labeling, fragmented cDNA was hybridized for 16 h at 45 °C on Affymetrix Rat Transcriptome Array 1.0 [transcript (gene) CSV version]. GeneChips were washed and stained in the Affymetrix Fluidics Station 450. All arrays were scanned using Affymetrix® GeneChip Command Console, which was installed in GeneChip® Scanner 3000 7 G. The gene expression data were analyzed with a robust multichip analysis (RMA) algorithm Affymetrix default analysis settings and global scaling as a normalization method. Values presented are log2 RMA signal intensity. Differentially expressed genes were identiﬁed based on the Student’s t-test to compare the two groups. The threshold set for up and downregulated genes was a fold change > 1.5 and a p-value < 0.05.

**Western blotting:** Cells were collected and lysed in lysis buffer (Thermo Fisher Scientiﬁc, Cat#89900) containing 1% Halt protease inhibitor (Thermo Fisher Scientiﬁc, Cat#87786) on ice for 30 min. Then the lysates were centrifuged at 12, 000 g for 20 min at 4 °C. The supernatant could be stored at -80 °C for further use. Protein quantiﬁcation was performed using the Pierce BCA protein assay kit (Thermo Fisher Scientiﬁc, Cat#23225). Absorbance at 595 nm was measured using the microplate plate reader (Bio-Rad). Equal amounts of protein (15–20 μg) were loaded with a mixation of 5X Sample Buffer (Solarbio, Cat#P1040) and boiled to 99 °C for 5–8 min. Proteins were run on 4%–12% or 10% sodium dodecyl sulfate-polyacrylamide for 60–90 min at 120V. According to the manufacturer's instructions, the protein was transferred for 60–90 min at 100 V to a nitrocellulose membrane (0.2 μm pore size; Millipore, Cat#ISEQ00010). Membranes were blocked in 5% skim milk in TBST (Solarbio, Cat#927-50100). All primary antibodies were used in a dilution of 1:1000 in 0.1%TBS-Tween. The membranes were incubated overnight at 4 °C. The membranes were washed three times in 0.1% TBS-Tween (Sigma-Aldrich, Cat#P9416). Secondary antibodies were added in a concentration of 1:5000 in 0.1% TBS-Tween and incubated for 2 h at room temperature. The membranes were washed three times in 0.1% TBS Tween. The manufacturer's instructions detected protein bands with an enhanced chemiluminescence western blotting detection kit (Thermo Fisher Scientiﬁc, Cat#34577).

**Measurement of intracellular protein content:** 5–10×10^4^ rTSPCs from young and aged rats with or without POG stimulation were centrifuged at 300 g for 10 min and then resuspended in 10 uL of radioimmunoprecipitation assay (RIPA) buffer (Thermo Fisher Scientific, Cat#89900). Harvested lysates (2 μL) were diluted in 8 μL of PBS, and the protein concentration was measured using the Pierce Protein Assay Kit (Thermo Fisher Scientific, Cat#23225) according to the manufacturer’s protocol. Absorbance at 595 nm was measured using the microplate plate reader (BioRad).

**ShRNA knockdown of ATG7**: Lentiviral expression vector pGLVH1/GFP+Puro-encoding shRNA (purchased from GenePharma) was used to infect aged rTSPCs from aged rats. The sequences of the shRNA target ATG7 and scrambled shRNA are provided in Supplementary Materials. After stable knockdown of ATG7, cells were stimulated by DMSO or POG as labeled in the legend. Then, cells in sh NC+POG, sh ATG7+DMSO and sh ATG7+POG groups were used for the following examinations.

**GFP-LC3 transfection:** 4×10^6^ rTSPCs from young and aged rats were seeded onto the 10-cm dish. Packaged adenovirus (Hanbio) was added to the culture medium. After 4 h of virus infection, cells were replaced with fresh medium with a final concentration of Pummvcin of 0.1 mg/L for resistance. For screening, the DMEM medium containing Puromycin was changed every three days. After six days of culture, infected cells grew normally. The expression of GFP protein in infected rTSPCs was observed by microscope. To examine autophagy activity in the status of normal (control) and starvation with chloroquine stimulation, cells were seeded onto a 24-well plate under different stimulus for 6–8 h.

**Oil Red O staining:** Briefly, 8 μm cryostat-cut sections were dried for 30 min at room temperature. In order to examine the lipid droplet formation, Oil Red O staining was performed according to the manufacturer's instructions with a kit (Solarbio, Cat#KT025). Image acquisition was performed with a Nikon microscope. Digital images were converted into 8-bit greyscale images. The staining was quantiﬁed using Image J software, measuring the proportion of lipid droplet deposition areas.

**Immunoﬂuorescence staining:** Cells were seeded onto 12 or 24-well plates with cell culture slides. After the required time, the cells were fixed in 4% paraformaldehyde for 15 min at room temperature. Subsequently, the cells were washed three times with PBS (5 min, shaking). If permeabilization, the cells were incubated with TBST (0.1% Triton X-100 in TBS) for 15 min. The samples were then blocked in TBST containing 5% normal donkey serum (NDS). Primary antibodies were diluted in TBST and incubated overnight at 4°C in a humidiﬁed chamber. Excess antibodies were washed off three times in TBST. Appropriate Alexa Fluor 488- and Alexa Fluor 594-conjugated secondary antibodies (Thermo Fisher Scientific, 1:400) diluted in TBST were used for 1.5 h at room temperature. Again, the excess antibody was washed off three times in TBST. Finally, nuclei were stained with DAPI (ZSGB-BIO, Cat#ZLI-9557) for 10 min in the dark. Confocal microscopic images were acquired with a Zeiss laser-scanning microscope 710 or a Leica TCS SP8 STED confocal microscope.

The harvested Achilles tendons were fixed immediately in 10% neutral buffered formalin for 24 h, dehydrated using gradient alcohol, and embedded in parafﬁn blocks for immunostaining tissues. Histological sections with 8 μm thickness were gathered using a microtome. Enzyme-activated antigen retrieval was performed by placing the paraformaldehyde-fixed samples in a 37 °C water bath for 45 minutes and cooling the slides at room temperature for 10 min. Then, sections were blocked in TBST with 5% NDS and 0.1% triton for 1 h and then were incubated overnight with primary antibodies. Primary antibodies used in this study were listed above. Non-immune immunoglobulins of the same isotype as the primary antibodies were used as a negative control. Next, appropriate Alexa Fluor 488- and Alexa Fluor 594-conjugated secondary antibodies (Thermo Fisher Scientific, 1:400) were utilized for 1.5 h at room temperature. After that, nuclei were stained with DAPI (ZSGB-BIO, Cat#ZLI-9557) for 10 min in the dark. Confocal microscopic images were acquired with a Zeiss laser-scanning microscope 710 or a Leica TCS SP8 STED confocal microscope.

**Supplemental Figures**

*
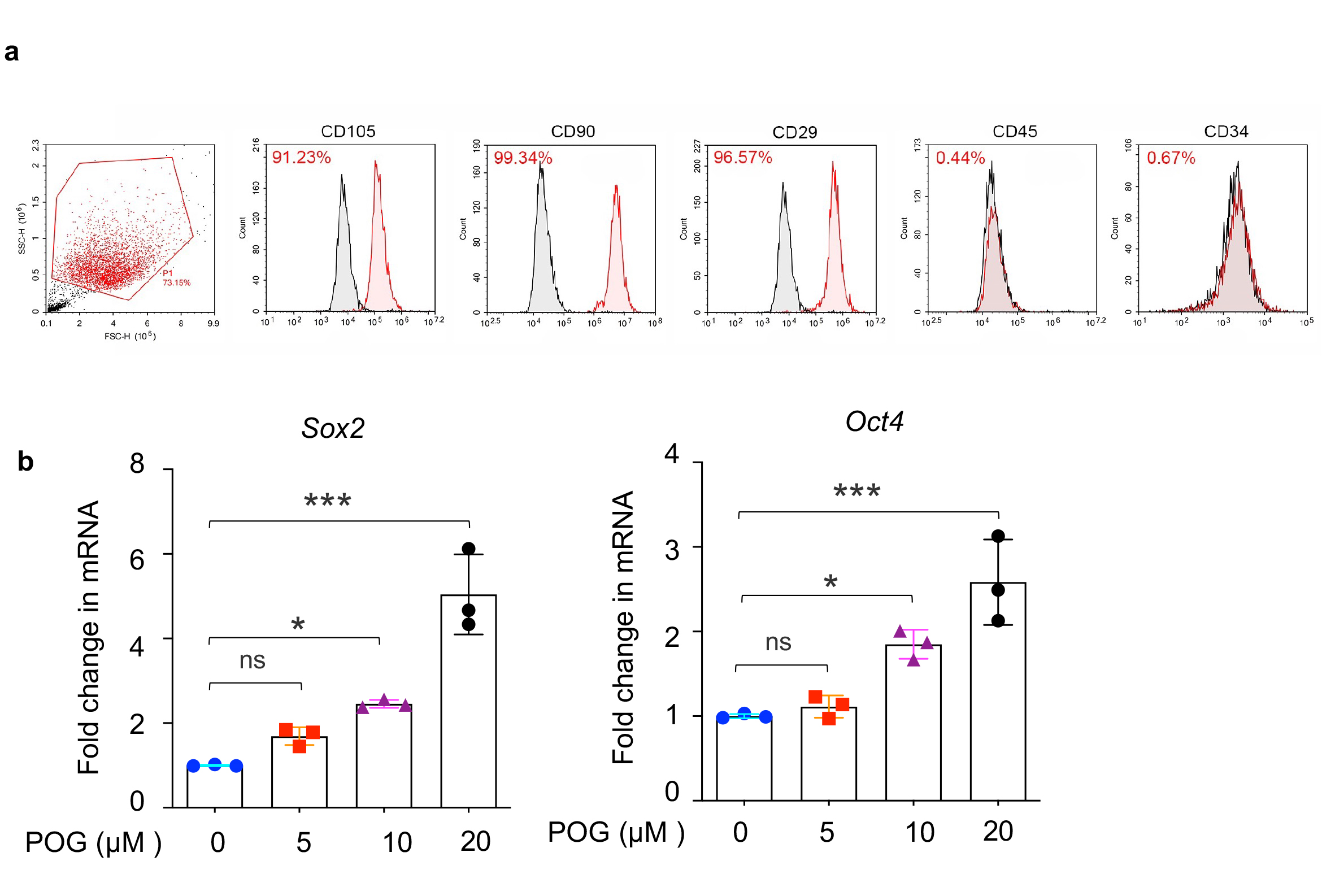
*

**Fig. S1.** **a** Flow cytometry analysis of the expression of cell surface markers in rTSPCs. **b** RT-qPCR of *Sox2* and *Oct-4* gene expression in rTSPCs at P12 with different concentrations of POG during serial passaging. (*n* = 3 biologically independent samples).

**
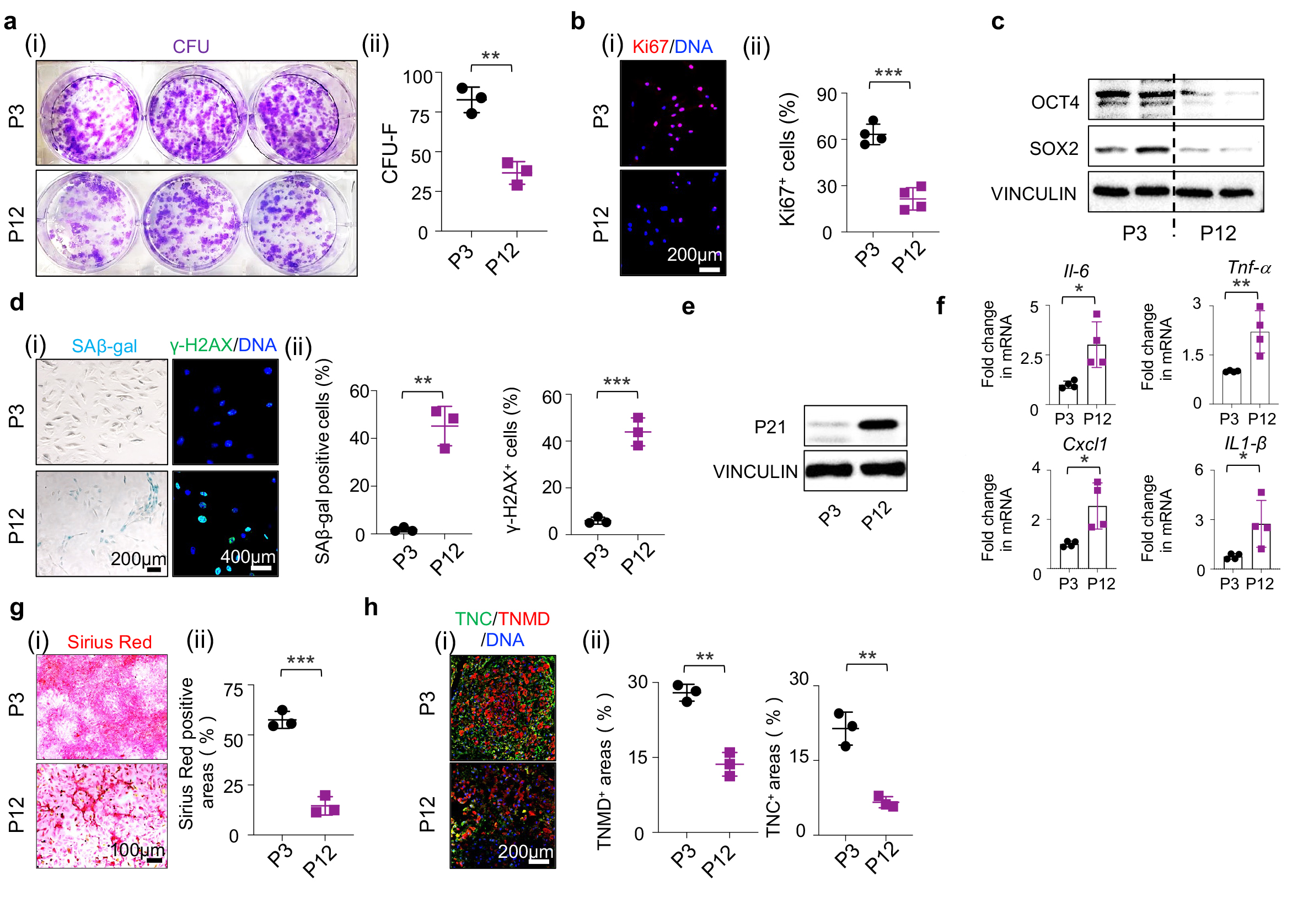
**

**Fig. S2.** *In vitro* long-term serial passaging impairs the stemness and tenogenic potential of rTSPCs. **a** (i) CFU-F assay of rTSPCs isolated from 3-month-old rats at P3 and P12 after serial passaging. (ii) Semi-quantification of (i) (*n* = 3 biologically independent samples). **b** (i) Immunofluorescence staining of Ki67 in rTSPCs at P3 and P12 after serial passaging. (ii) Semi-quantification of (i) (*n* = 4 biologically independent samples). **c** Western blotting of OCT4 and SOX2 in rTSPCs at P3 and P12 after serial passaging. **d** (i) SAβ-gal staining (left panel, blue cells are senescent cells) and immunofluorescence staining of DNA injury-related protein γ-H2AX (right panel) of rTSPCs at P3 and P12 after serial passaging. (ii) Semi-quantification of (i) (*n* = 3 biologically independent samples). **e** Western blotting of senescence-related protein P21 in rTSPCs at P3 and P12 after serial passaging. **f** RT-qPCR of inflammation-related genes *Il-6, Tnf-a, Cxcl1,* and *Il-1β* (*n* = 4 biologically independent samples). **g** (i) Sirius Red staining (pink areas are positively stained respectively) of TSPCs at P3 and P12 after 14 d of tenogenic induction. (ii) Semi-quantification of (i) (*n* = 3 biologically independent samples). **h** (i) Immunofluorescence staining of tenogenic markers TNC and TNMD in TSPCs at P3 and P12 after 14 d of tenogenic induction. (ii) Semi-quantification of (i) (*n* = 3 biologically independent samples). Data are represented as mean ± SD. (* *p* < 0.05; ** *p* < 0.01; *** *p* < 0.001)


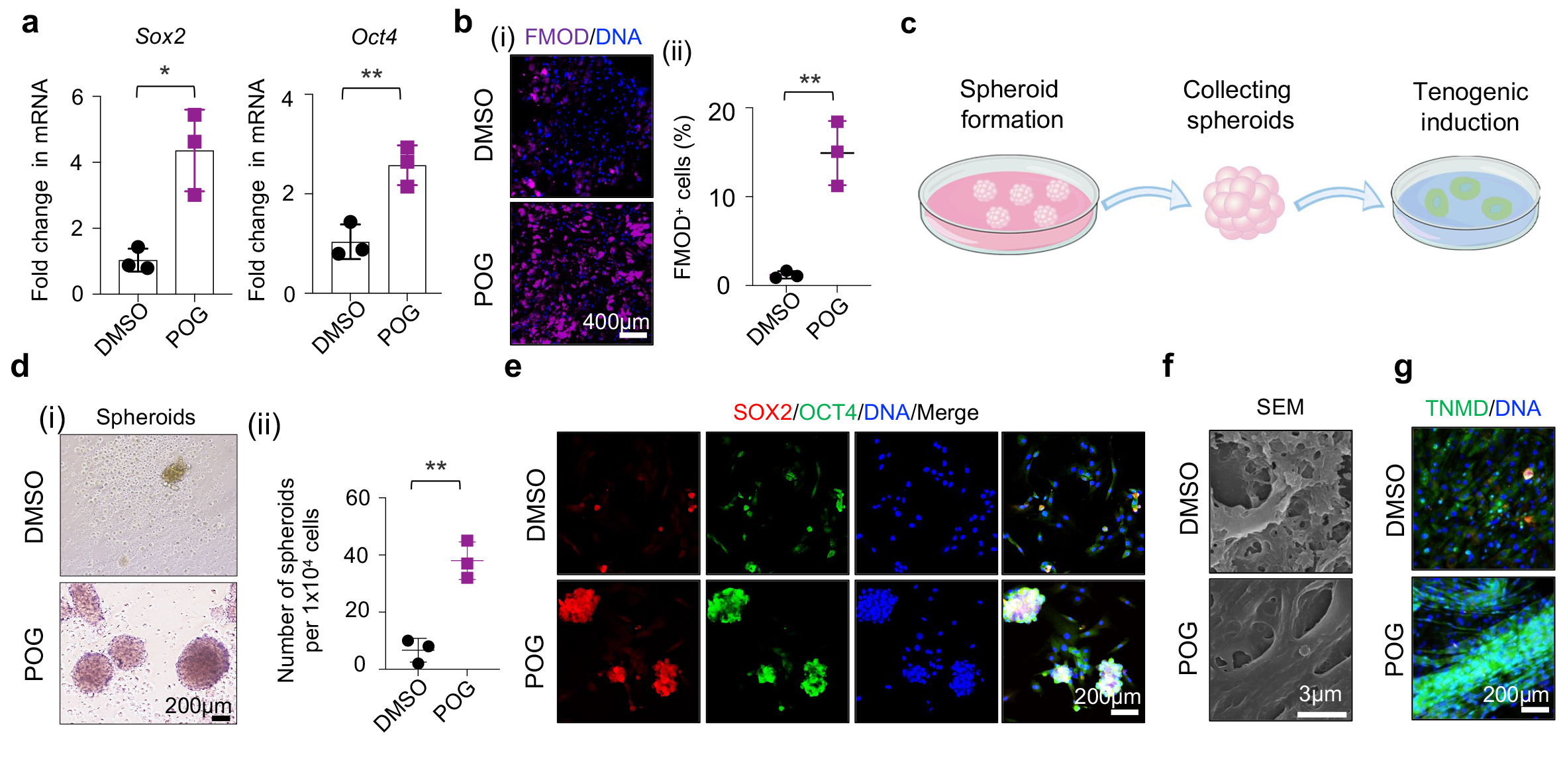


**Fig. S3.** POG treatment maintains rTSPC stemness and inhibits its senescence during *in vitro* serial passaging. **a** RT-qPCR of *Sox2* and *Oct-4* gene expression in rTSPCs at P12 with DMSO or POG treatment during serial passaging. (*n* = 3 biologically independent samples). **b** (i) Immunofluorescence staining (right panel) of the tenogenic marker FMOD of rTSPCs at P12 with DMSO or POG treatment after 14 d of tenogenic induction. (ii) Semi-quantification of (i) (*n* = 3 biologically independent samples). **c** Schematic outlining three-dimensional spheroid formation of rTSPCs at the 12th passage and subsequent tenogenic differentiation. **d** (i) Microscopic images of three-dimensional spheroids of rTSPCs at P12 with DMSO or POG treatment during serial passaging. (ii) Semi-quantiﬁcation of (i) (*n* = 3 biologically independent samples). **e** Immunoﬂuorescence staining of SOX2 and OCT4 in rTSPC spheroids at P12 with DMSO or POG treatment. **f** SEM microstructure of collagen ﬁbrils in rTSPC spheroids at P12 with DMSO or POG treatment after 21d of tenogenic induction. **g** Immunoﬂuorescence staining of TNMD in rTSPC spheroids at P12 with DMSO or POG treatment after 21-d tenogenic induction. Data are represented as mean ± SD. (* *p* < 0.05; ** *p* < 0.01)


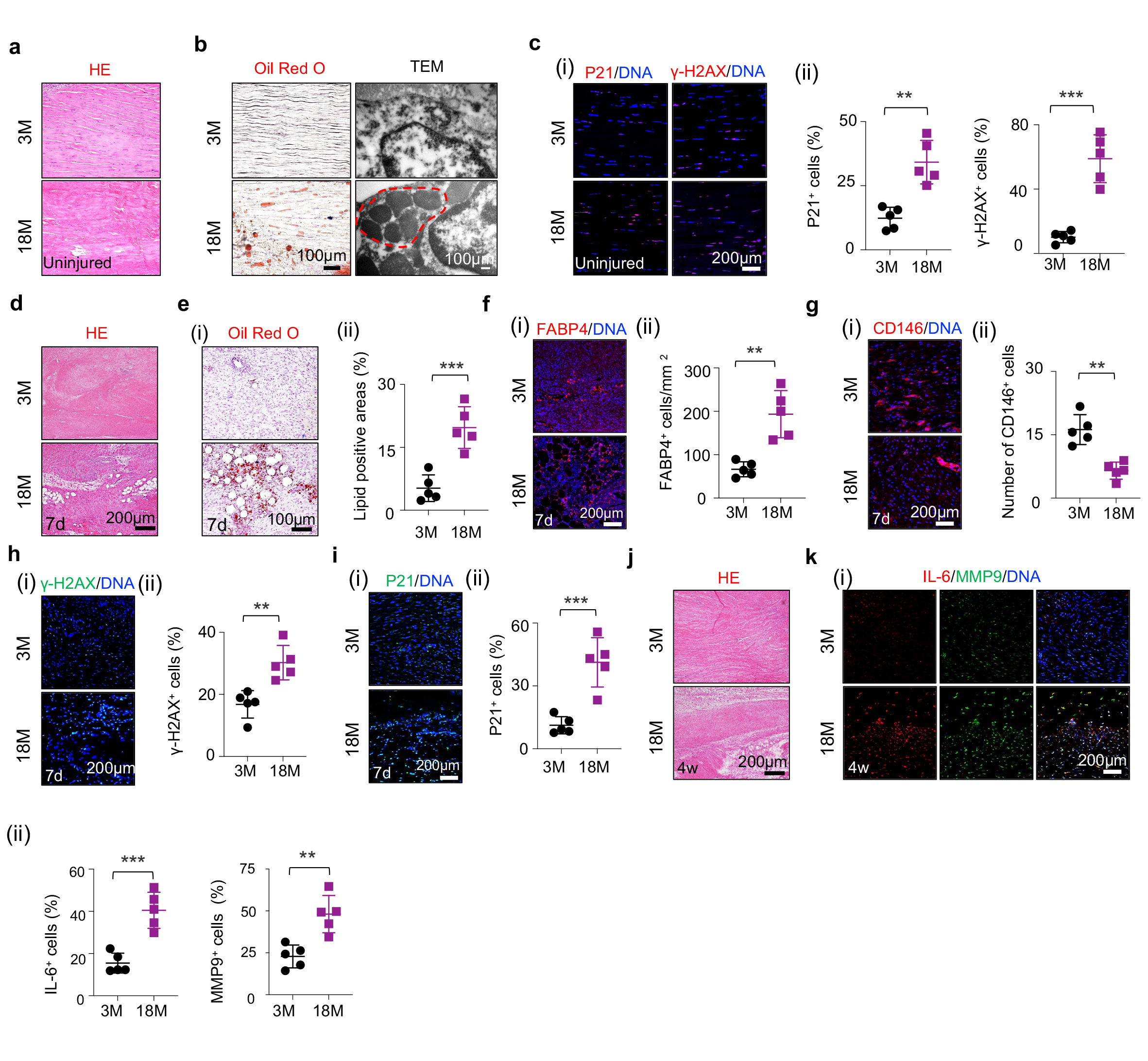
**Fig. S4.** Tendons from aged rats show accumulation of senescent cells, lipid deposition, and impaired tendon repair. **a** HE staining of uninjured Achilles tendons from the 3-month-old (3M) young and 18-month-old (18M) aged rats (*n* = 5 rats per group). **b** Oil Red O staining and TEM of uninjured Achilles tendons from the young and aged rats (*n* = 5 rats per group). **c** (i) Immunofluorescence staining of P21 and γ-H2AX in the uninjured Achilles tendons from the young and aged rats. (ii) Semi-quantification of (i) (*n* = 5 rats per group). **d** HE staining of injured Achilles tendons from the young and aged rats at 1 wk postoperatively. (ii) Semi-quantification of (i) (*n* = 5 rats per group). **e** Oil Red O staining of injured Achilles tendons from the young and aged rats at 1 wk postoperatively. (ii) Semi-quantification of (i) (*n* = 5 rats per group). **f** (i) Immunofluorescence staining of FABP4 positive cells in injured Achilles tendons from the young and aged rats at 1 wk postoperatively. (ii) Semi-quantification of (i) (*n* = 5 rats per group). **g** (i) Immunofluorescence staining of CD146 in injured Achilles tendons from the young and aged rats at 1 wk postoperatively. (ii) Semi-quantification of (i) (*n* = 5 rats per group)*.* **h** (i) Immunofluorescence staining of γ-H2AX in injured Achilles tendons from the young and aged rats at 1 wk postoperatively. (ii) Semi-quantification of (i) (*n* = 5 rats per group). **i** (i) Immunofluorescence staining of the senescent marker P21 in injured Achilles tendons from the young and aged rats at 1 wk postoperatively. (ii) Semi-quantification of (i) (*n* = 5 rats per group). **j** HE staining of injured Achilles tendons from the young and aged rats at 4 wk postoperatively (*n* = 5 rats per group). **k** (i) Immunofluorescence staining of inflammation-related markers IL-6 and MMP9 of in injured Achilles tendons from the young and aged rats at 4 wk postoperatively. (ii) Semi-quantification of (i) (*n* = 5 rats per group). Data are represented as mean ± SD. (** *p* < 0.01; *** *p* < 0.001)

**
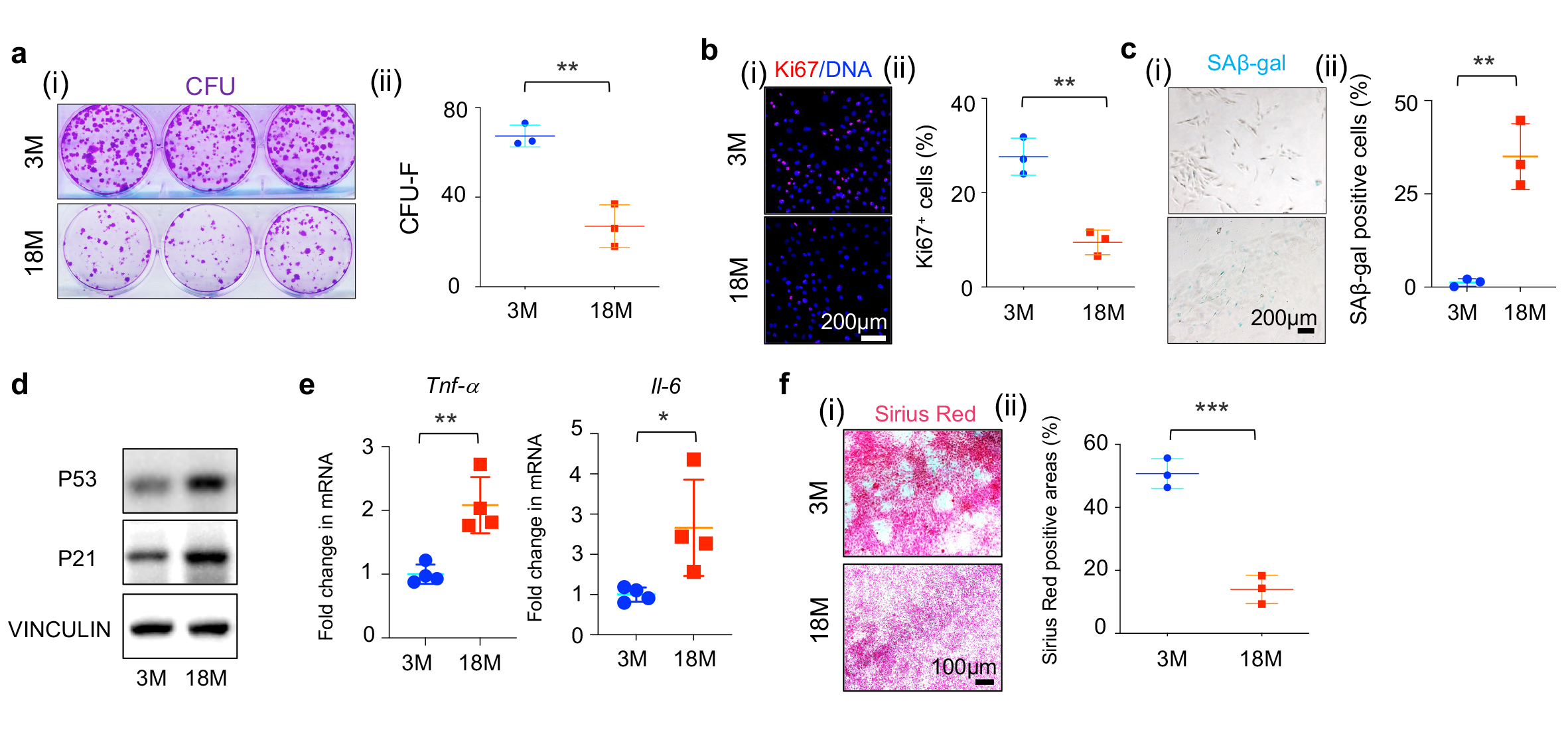
Fig. S5.** Aged rTSPCs isolated from aged rats display declined stemness and tenogenic potential due to cellular senescence. **a** (i) CFU-F assay of rTSPCs isolated from the young and aged rats. (ii) Semi-quantification of (i) (*n* = 3 biologically independent samples). **b** (i) Immunofluorescence staining of Ki67 in rTSPCs isolated from the young and aged rats. (ii) Semi-quantification of (i) (*n* = 3 biologically independent samples). **c** (i) SAβ-gal staining (left panel) of rTSPCs isolated from the young and aged rats. Blue cells are senescent cells. (ii) Semi-quantification of (i) (*n* = 3 biologically independent samples). **d** Western blotting of senescence-related proteins P21 and P53 in rTSPCs isolated from the young and aged rats. **e** RT-qPCR of *Il-6* and *Tnf-a* gene expression in rTSPCs isolated from the young and aged rats (*n* = 3 biologically independent samples). **f** (i) Sirius Red staining of rTSPCs isolated from the young and aged rats after 14 d of tenogenic induction. (ii) Semi-quantification of (i) (*n* = 3 biologically independent samples). Data are represented as mean ± SD. (* *p* < 0.05; ** *p* < 0.01; *** *p* < 0.001)


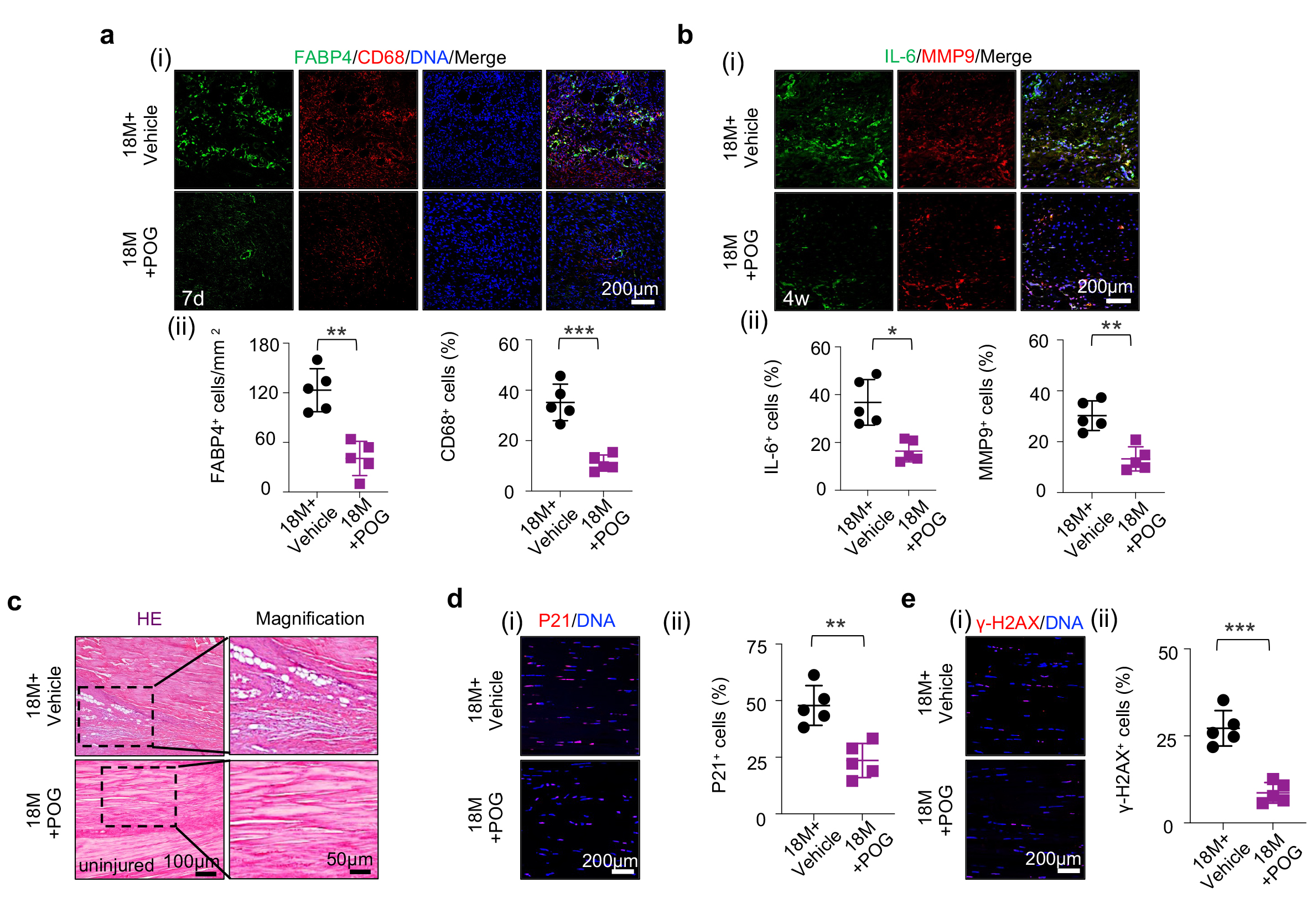


**Fig. S6.** Long-term oral administration of POG delays tendon degeneration and promotes aged tendon self-repair in partial transection tendon injuries. **a** (i) Immunofluorescence staining of FABP4 and CD68 in injured Achilles tendons from 18-month-old rats with vehicle administration (18M+Vehicle) and 18-month-old rats with POG administration (18M+POG) at 1 wk postoperatively*.* (ii) Semi-quantification of (i) (*n* = 5 rats per group). **b** (i) Immunofluorescence staining of IL-6 and MMP9 in injured Achilles tendons from the 18M+Vehicle and 18M+POG groups at 4 wk postoperatively*.* (ii) Semi-quantification of (i) (*n* = 5 rats per group). **c** HE staining of uninjured Achilles tendons from the 18M+Vehicle and 18M+POG groups at 1 wk postoperatively (*n* = 5 rats per group). **d** (i) Immunofluorescence staining of P21 in uninjured Achilles tendons from the 18M+Vehicle and 18M+POG groups at 1 wk postoperatively*.* (ii) Semi-quantification of (i) (*n* = 5 rats per group). **e** (i) Immunofluorescence staining of γ-H2AX in uninjured Achilles tendons from the 18M+Vehicle and 18M+POG groups at 1 wk postoperatively*.* (ii) Semi-quantification of (i) (*n* = 5 rats per group). Data are represented as mean ± SD. (* *p* < 0.05; ** *p* < 0.01; *** *p* < 0.001)


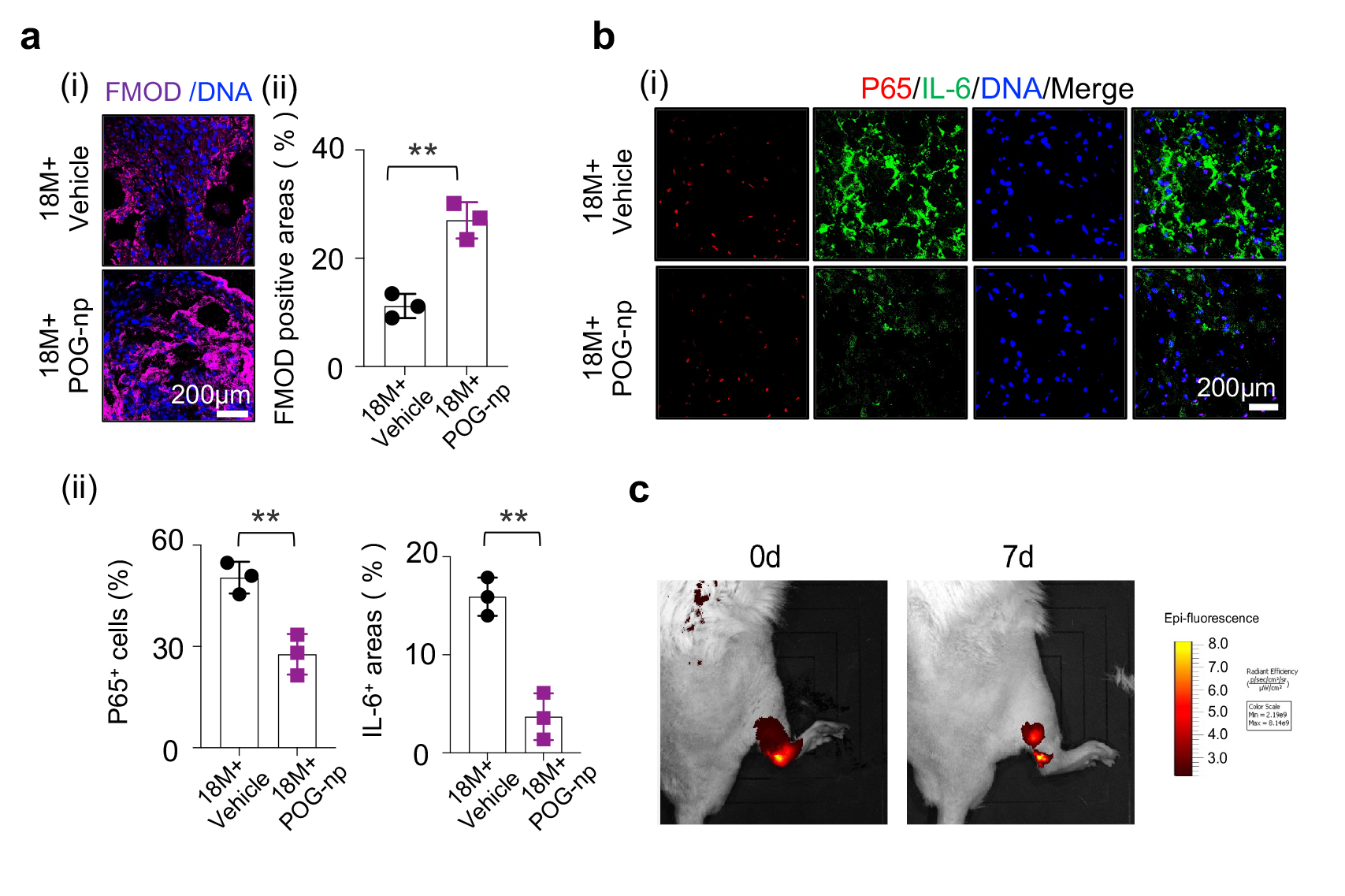


**Fig. S7.** PLGA-loaded-POG nanoparticles enhance rTSPC stemness and rejuvenate senescent phenotypes. **a** (i) Immunofluorescence staining of the tenogenic marker FMOD of vehicle- and POG-nps-treated aged rTSPCs isolated from the aged rats after 14 d of tenogenic differentiation. (ii) Semi-quantification of (i) (*n* = 3 biologically independent samples). **b** (i) Immunofluorescence staining of tenogenic markers IL-6 and P65 of Vehicle- and POG-nps-treated aged rTSPCs after 14 d of tenogenic differentiation. (ii) Semi-quantification of (i) (*n* = 3 biologically independent samples).**c** *In vivo* fluorescence images of the injured Achilles tendon after subcutaneous injection of PLGA nanoparticles. Data are represented as mean ± SD. (** *p* < 0.01)


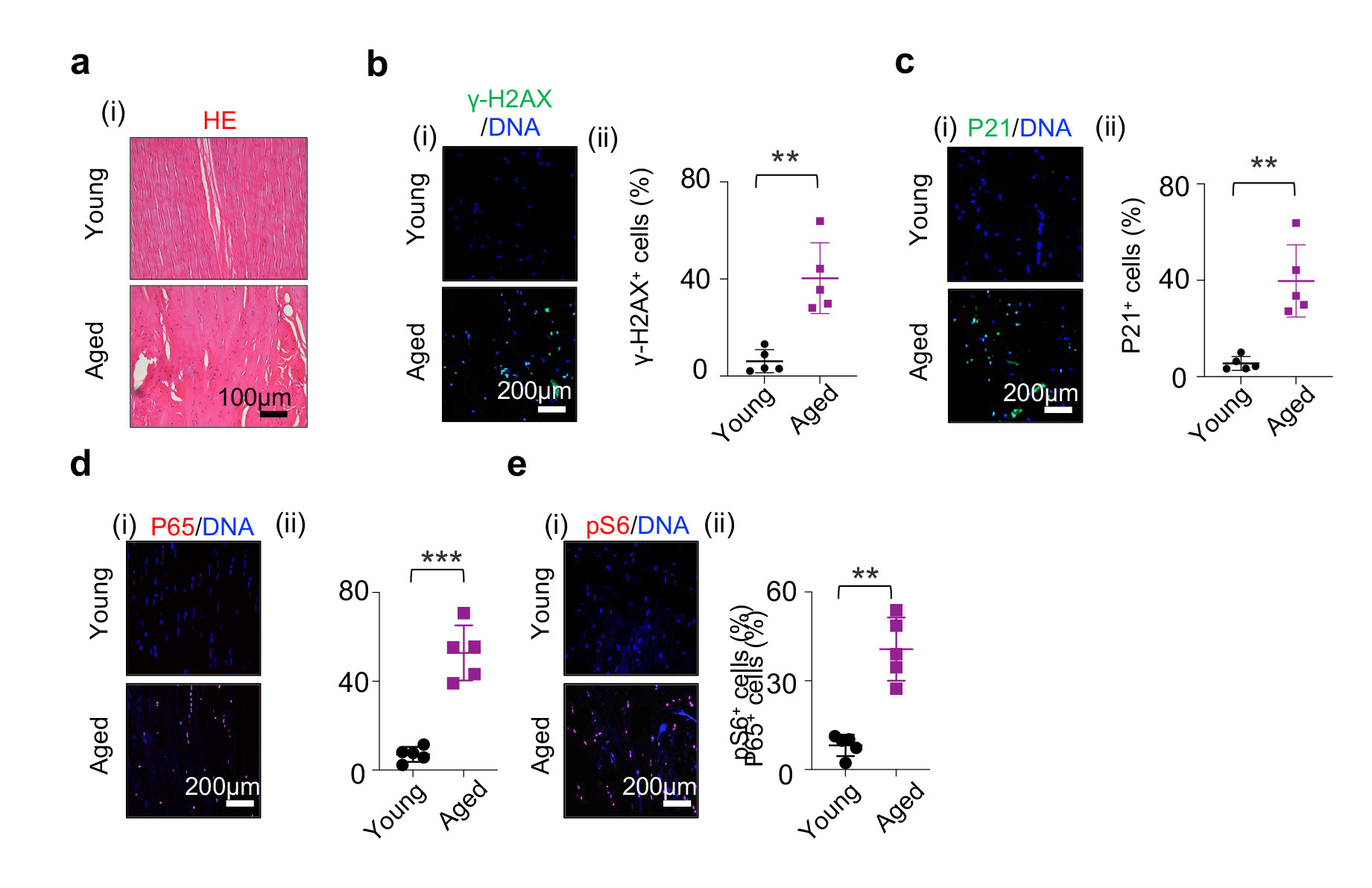


**Fig. S8.** Human tendons from middle-aged individuals display obvious senescence. **a** (i) HE staining of normal tendons from the young adult and middle-aged individuals. **b** (i) Immunofluorescence staining of γ-H2AX in normal tendons from the young adult and middle-aged individuals. (ii) Semi-quantification of (i) (*n* = 5 biologically independent samples). **c** (i) Immunofluorescence staining of P21 in normal tendons from the young adult and middle-aged individuals. (ii) Semiquantification of (i) (*n* = 5 biologically independent samples). **d** (i) Immunofluorescence staining of P65 in normal tendons from the young adult and middle-aged individuals. (ii) Semi-quantification of (i) (*n* = 5 biologically independent samples). **e** (i) Immunofluorescence staining of pS6 in normal tendons from the young adult and middle-aged individuals. (ii) Semi-quantification of (i) (*n* = 5 biologically independent samples). Data are represented as mean ± SD. (** *p* < 0.01; *** *p* < 0.001)

**
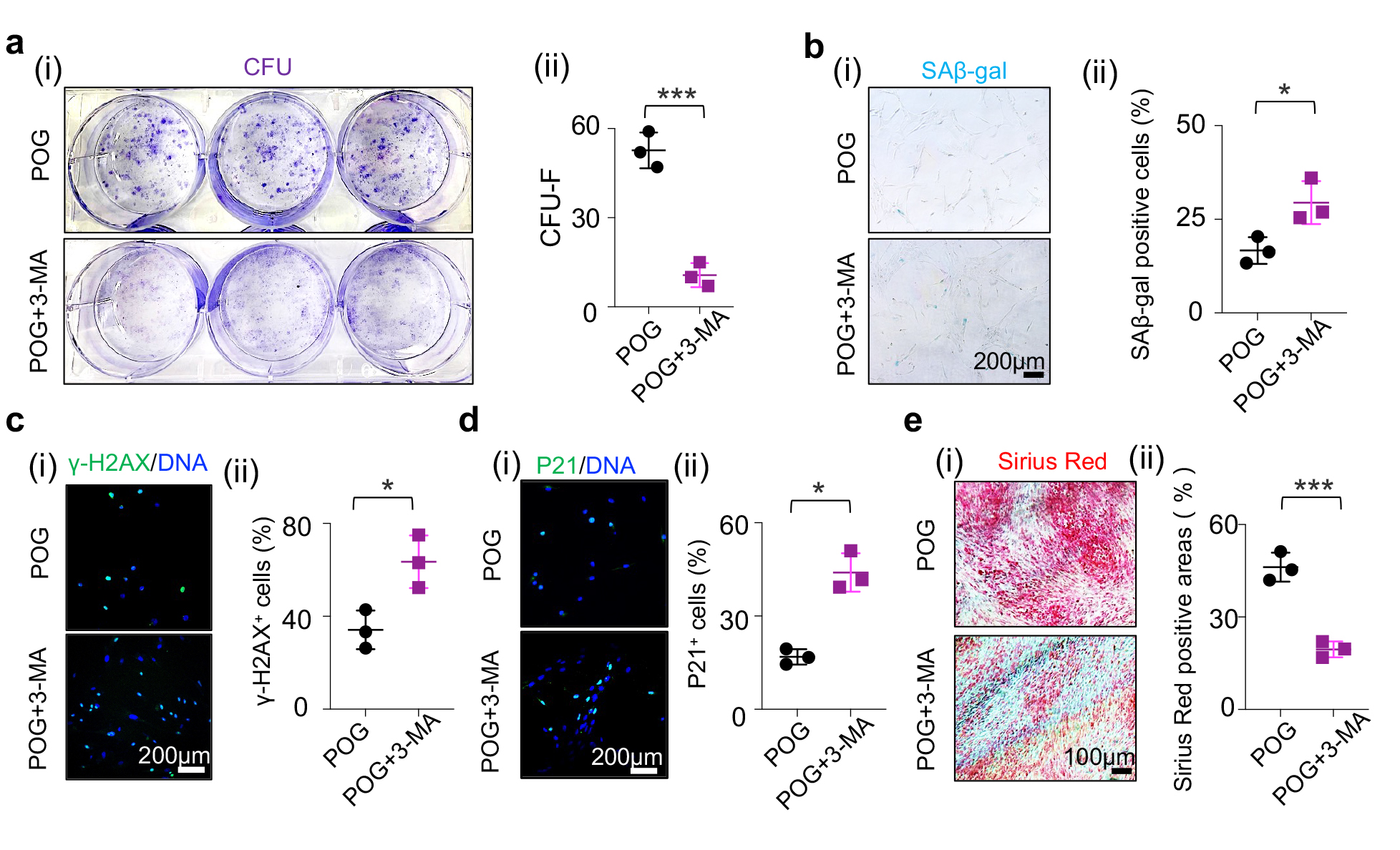
**

**Fig. S9.** The rejuvenation effect of POG on senescent hTSPCs is counteracted by the intervention of 3-MA. **a** (i) CFU-F assay of POG-treated and POG&3MA-treated aged hTSPCs. (ii) Semi-quantification of (i) (*n* = 3 biologically independent samples). **b** (i) SAβ-gal staining of POG-treated and POG&3MA-treated aged hTSPCs. (ii) Semi-quantification of (i) (*n* = 3 biologically independent samples). **c** (i) Immunofluorescence staining of γ-H2AX in POG-treated and POG&3MA-treated aged hTSPCs. (ii) Semi-quantification of (i) (*n* = 3 biologically independent samples). **d** (i) Immunofluorescence staining of P21 in POG-treated and POG&3MA-treated aged hTSPCs. (ii) Semi-quantification of (i) (*n* = 3 biologically independent samples). **e** (i) Sirius Red staining of POG-treated and POG&3MA-treated aged hTSPCs. (ii) Semi-quantification of (i) (*n* = 3 biologically independent samples). Data are represented as mean ± SD. (* *p* < 0.05; *** *p* < 0.001)

**
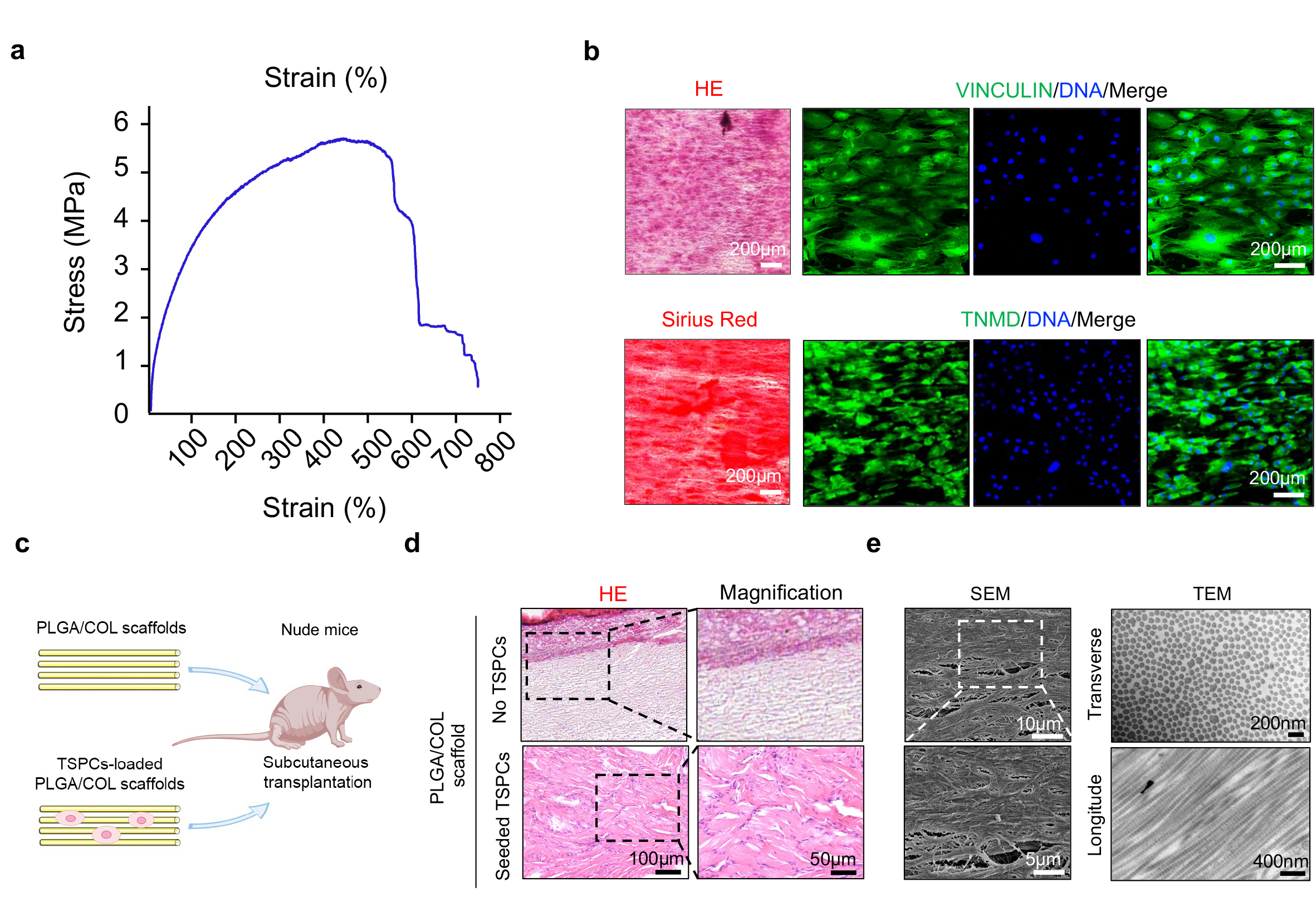
**

**Fig. S10.** Parallel PLGA/COL scaffolds possess good tenogenic-induction capacity. **a** A representative axial stress-strain curve of PLGA/COL scaffolds. **b** HE, Sirius Red, and immunofluorescence staining of VINCULIN and TNMD in aged rTSPCs cultured on PLGA/COL scaffolds after 14 d of tenogenic induction. **c** Schematic outlining transplantation of PLGA/COL scaffolds with and without rTPSCs into athymic mice for 8 wk. **d** HE staining of neotissues. *n* = 5 mice per group. **e** SEM and TEM of collagen pattern of neotissues from the mice seeded with rTSPCs.


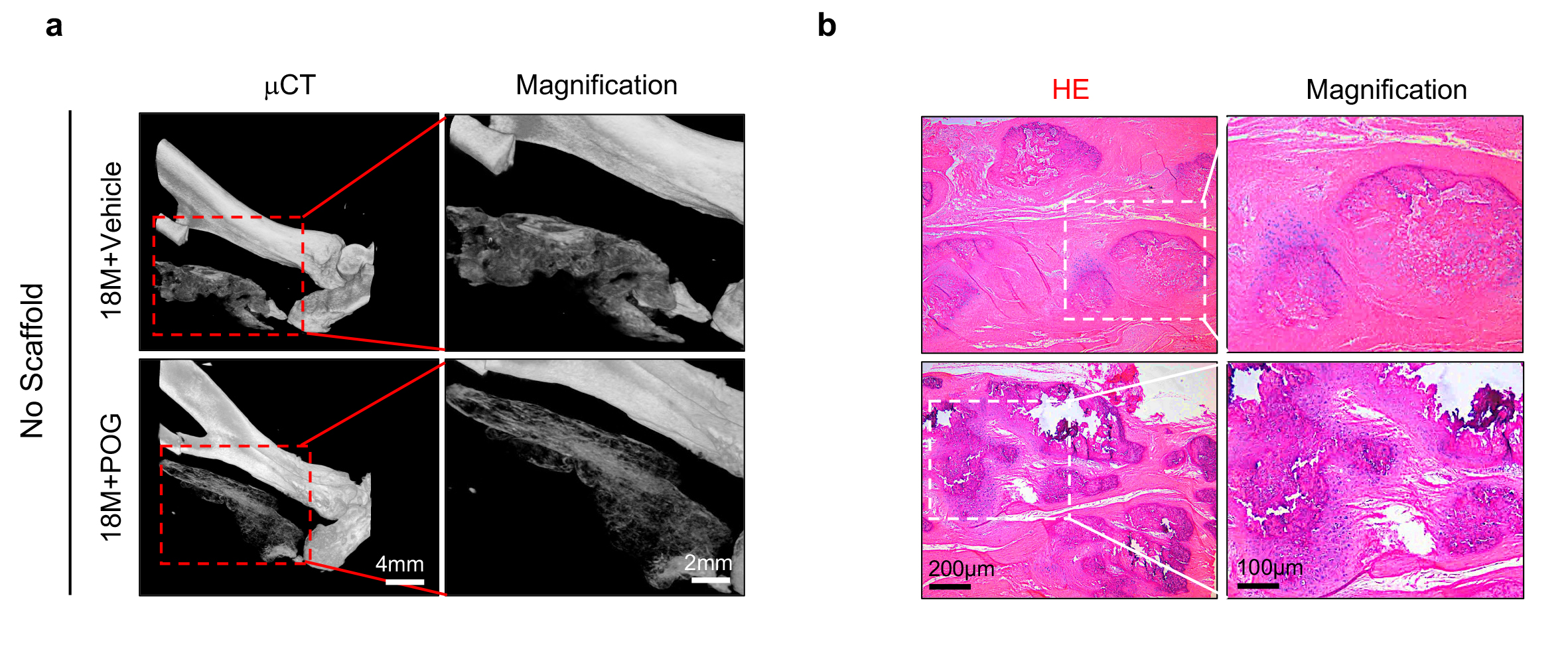


**Fig. S11.** Full-cut window tendon defects in aged rats heal poorly and result in large-scale heterotopic ossification without the transplantation of scaffolds. **a** µCT scans of regenerated Achilles tendons from the 18M+Vehicle and 18M+POG groups without transplantation of PLGA/COL scaffolds at 8 wk postoperatively. *n* *=* 5 rats per group*.* **b** HE staining of neotissues from the 18M+Vehicle and 18M+POG groups without transplantation of PLGA/COL scaffolds at 8 wk postoperatively. *n* *=* 5 rats per group*.*

**
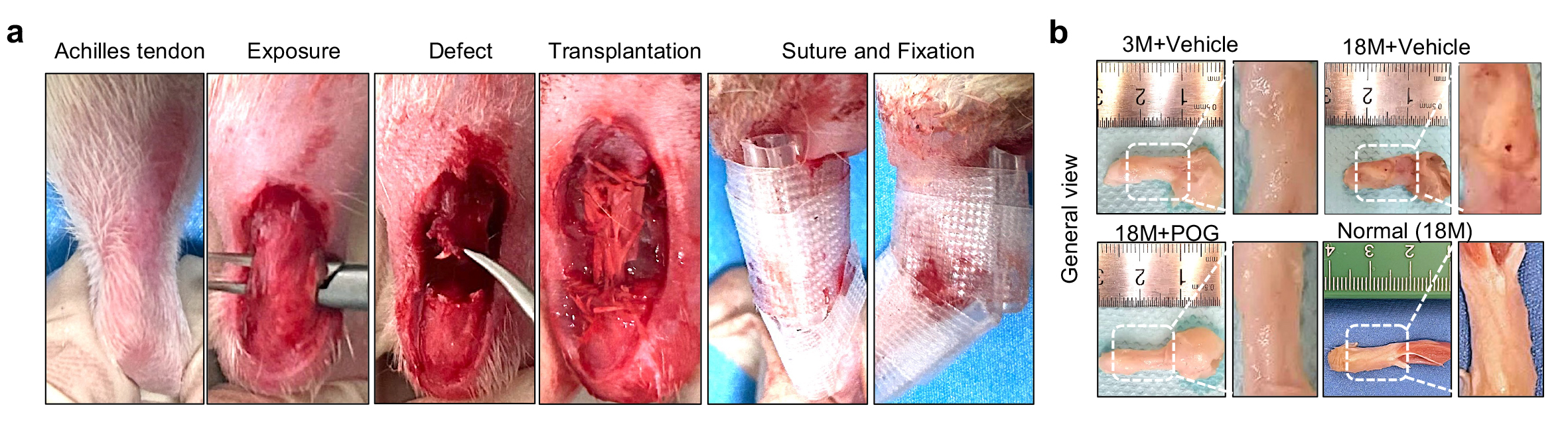
**

**Fig. S12**. Gross morphology of neotendons in full-cut tendon ruptures by POG administration combining the transplantation of biomimetic scaffolds in aged rats. **a** Schematic outlining surgical procedure of full-cut tendon window defect in rats. **b** Representative gross morphology of regenerated Achilles tendons of each group at 8 wk postoperatively. *n =* 5 rats per group.


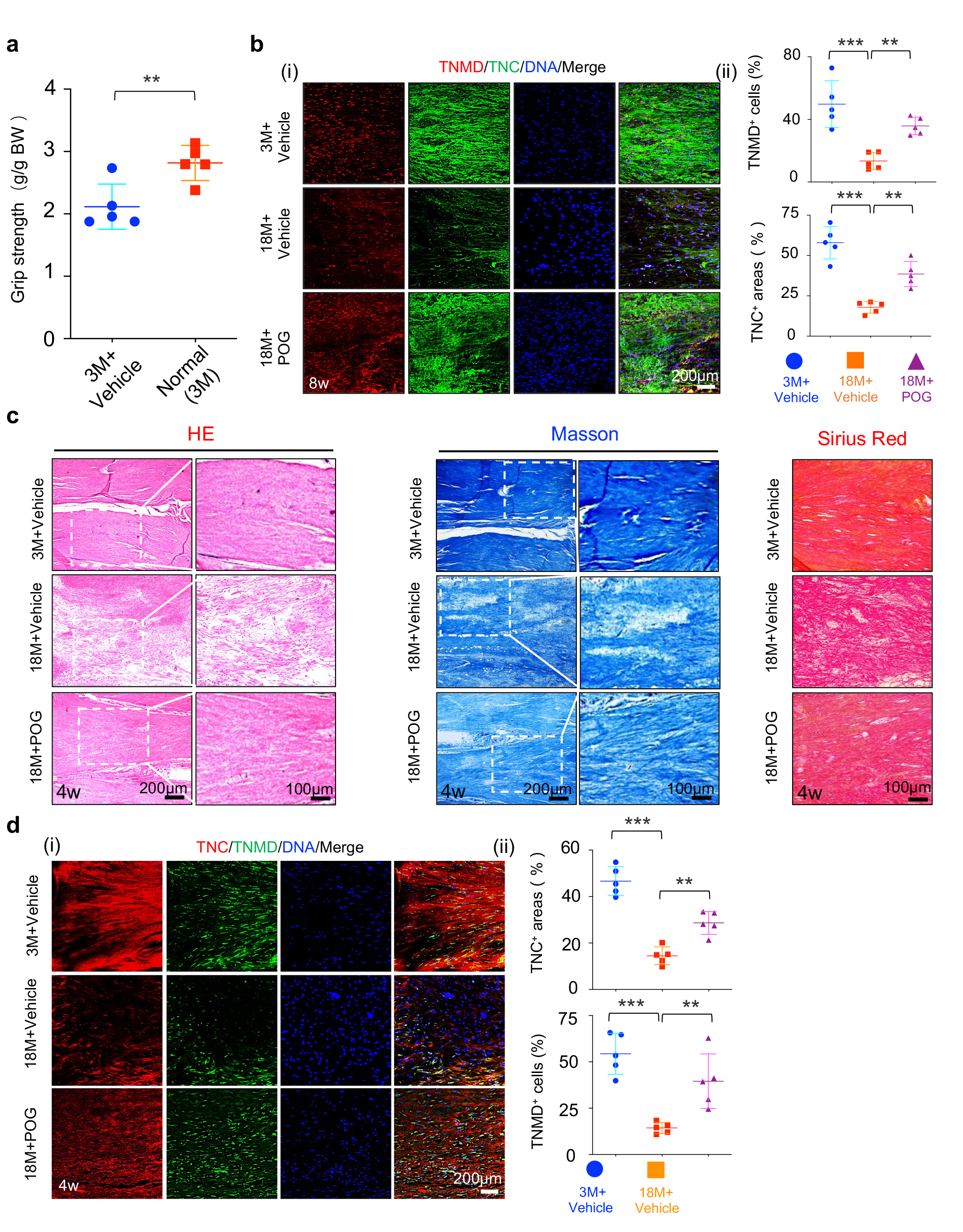


**Fig. S13.** Regeneration of full-cut tendon ruptures by POG administration combining the transplantation of biomimetic scaffolds in aged rats. **a** Grip strength of the normal rats and the rats in the 3M+Vehicle group transplanted with PLGA/COL scaffolds at 8 wk postoperatively. Normal (3M): Normal uninjured tendons from the young rats (*n* *=* 5 rats per group). **b** (i) Immunofluorescence staining of TNC and TNMD of sections from the 3M+Vehicle, 18M+Vehicle and 18M+POG groups at 8 wk postoperatively. (ii) Semi-quantification of (i) (*n =* 5 rats per group). **c** HE, Masson’s trichrome, and Sirius Red staining of neotendons from different groups at 4 wk postoperatively. *n* *=* 5 rats per group*.* **d** (i) Immunofluorescence staining of TNC and TNMD of neotendons from each group at 4 wk postoperatively. (ii) Semi-quantification of (i) (*n* = 5 rats per group). Data are represented as mean ± SD. (** *p* < 0.01; *** *p* < 0.001)


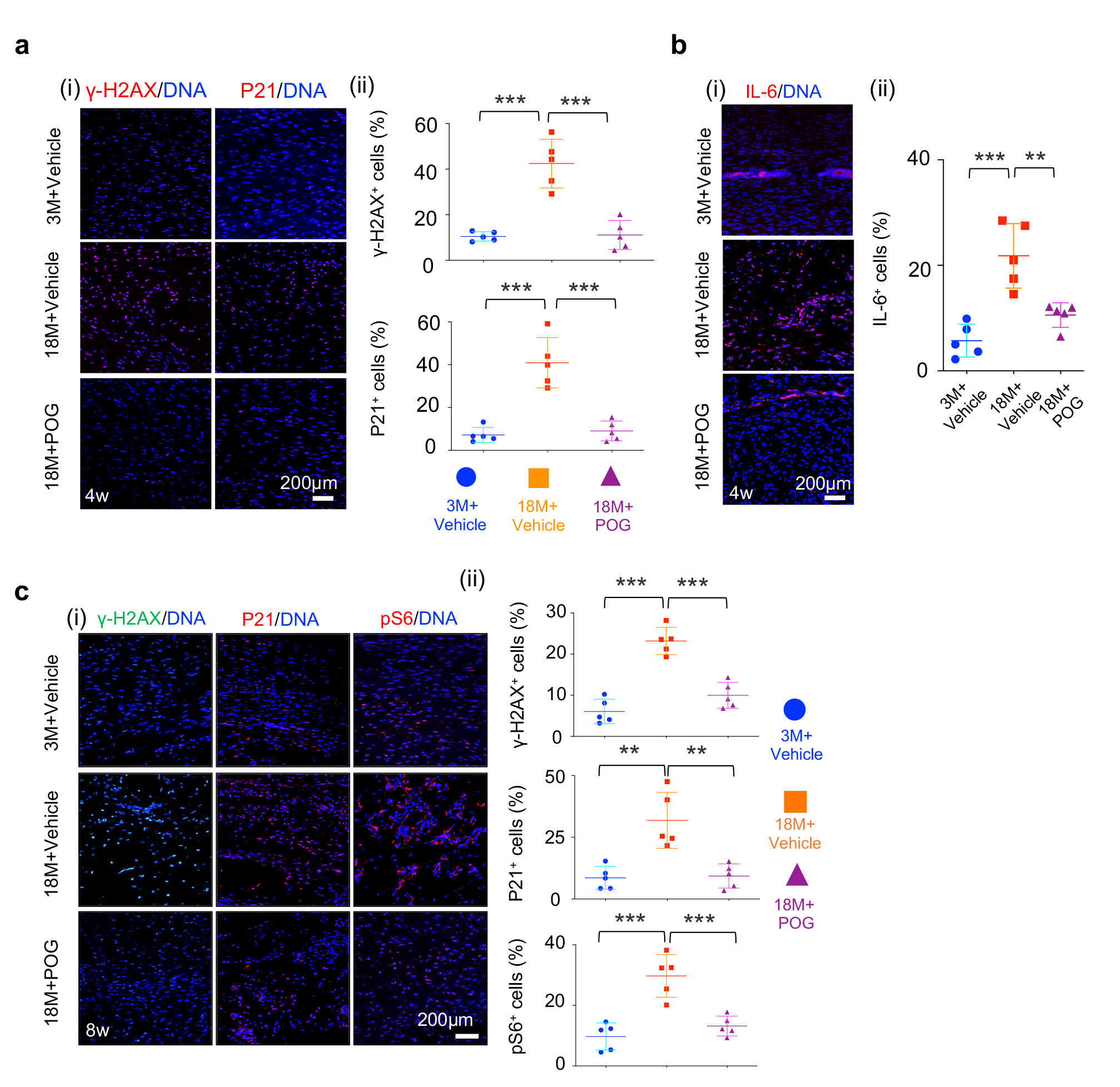


**Fig. S14.** Functional and structural regeneration of full-cut tendons by POG administration combining the transplantation of biomimetic scaffolds in aged rats. **a** (i) Immunofluorescence staining of γ-H2AX and P21 in neotendons from each group at 4 wk postoperatively. (ii) Semi-quantification of (i) (*n* = 5 rats per group). **b** (i) Immunofluorescence staining of IL-6 in neotendons from each group at 4 wk postoperatively. (ii) Semi-quantification of (i) (*n* = 5 rats per group). **c** (i) Immunofluorescence staining of γ-H2AX, P21, and pS6 of sections from the 3M+Vehicle, 18M+Vehicle, and 18M +POG groups at 8 wk postoperatively. (ii) Semi-quantification of (i) (*n =* 5 rats per group). Data are represented as mean ± SD. (** *p* < 0.01; *** *p* < 0.001)

**
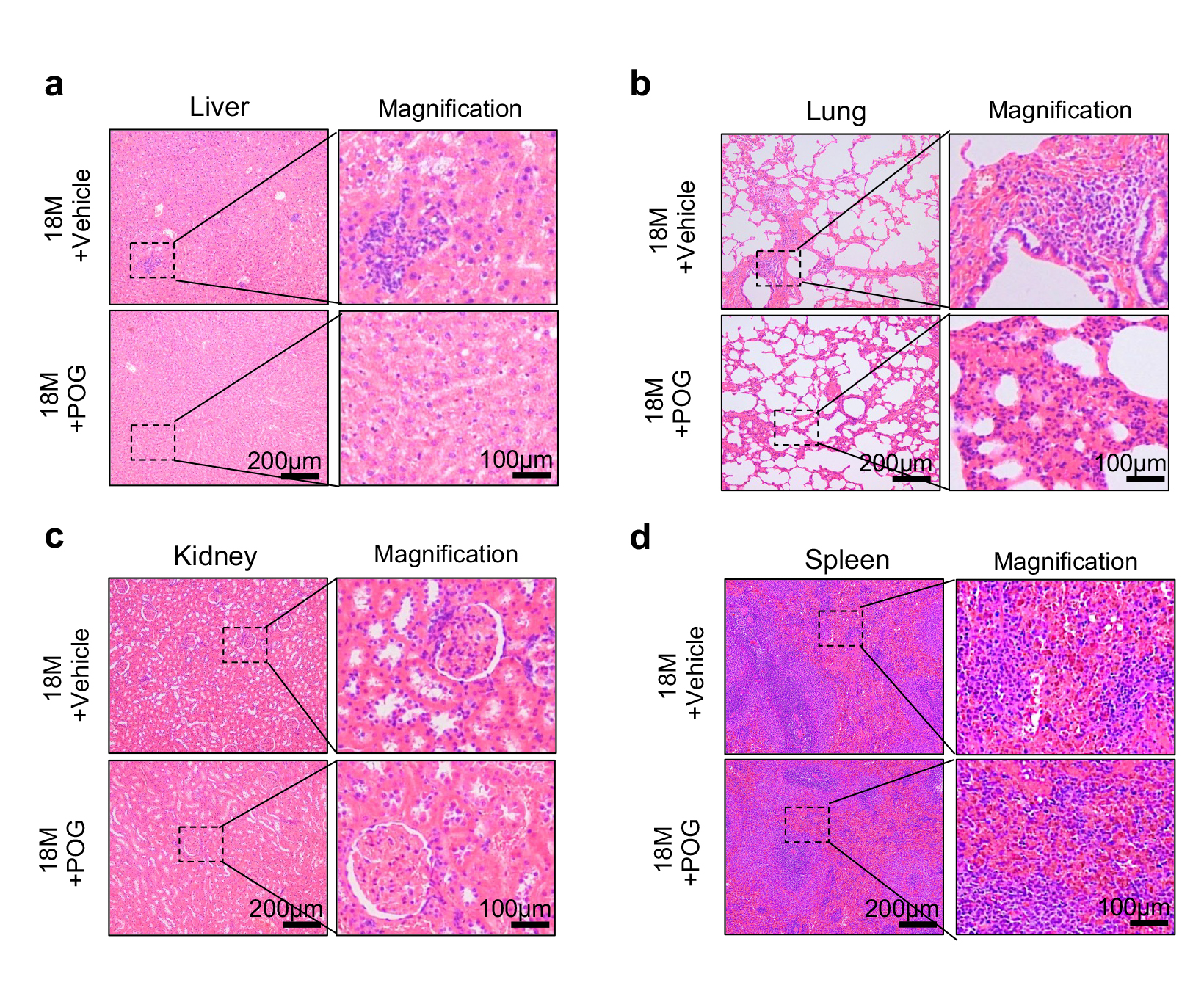
**

**Fig. S15.** Two-month oral administration of POG in aged rats presents no toxicity on liver, lung, spleen, and kidney. (a) – (b) HE staining of liver (a), lung (b), kidney (c) and spleen (d) from the aged rats after 2-month oral administration of vehicle or POG (*n* *=* 5 rats per group).

**Table S1**. List of top-ranked molecules by DLEPS.

| **Index** | **ID** | **Name** | **CAS** | **cs_rat tendon** | **cs_Yamanaka Factor** |
| --- | --- | --- | --- | --- | --- |
| 683 | T5S0733 | Picroside III | 64461-95-6 | 0.200797631 | 0.634977287 |
| 1488 | T5S1598 | Mulberroside C | 102841-43-0 | 0.288022898 | 0.553049968 |
| 825 | S7218 | Alvelestat (AZD9668) | 848141-11-7 | 0.175038818 | 0.546966256 |
| 324 | TWS0373 | Glyasperin F |  | 0.1945923 | 0.530012979 |
| 2080 | T6S2271 | Vindoline | 2182-14-1 | 0.297731112 | 0.518494484 |
| 1259 | T3S1363 | Agnuside | 11027-63-7 | 0.191947322 | 0.467850963 |
| 1665 | T4S1820 | Parishin A | 62499-28-9 | 0.263523848 | 0.446868916 |
| 1033 | T4S1114 | Dryocrassin ABBA | 12777-70-7 | 0.215857979 | 0.401497945 |
| 1541 | T7S1680 | Prim-O-  glucosylcimifugin | 80681-45-4 | 0.173152018 | 0.376784555 |
| 719 | TMS0774 | Sophorabioside | 2945-88-2 | 0.299812765 | 0.367834739 |
| 1311 | T4S1422 | Praeruptorin E | 78478-28-1 | 0.222020508 | 0.351800779 |
| 1258 | TCS1368 | Vicenin -1 | 35927-38-9 | 0.23624386 | 0.332630327 |

**Table S2.** List of reagents or resources used in the study.

| **REAGENT or RESOURCE** | **SOURCE** | **IDENTIFIER** |
| --- | --- | --- |
| **Antibodies** | | |
| Rabbit monoclonal anti-Ki67 | Abcam | Cat# AB16667 |
| Rat monoclonal anti-Ki67 | Ebioscience | Cat#14-5698-82 |
| Goat polyclonal anti-Oct4 | Abcam | Cat# AB27985 |
| Rabbit polyclonal anti-Oct4 | Abcam | Cat# AB18976 |
| Rabbit polyclonal anti-Sox2 | Abcam | Cat#AB97959 |
| Mouse monoclonal anti-Sox2 | Abcam | Cat#AB79351; |
| Rabbit polyclonal anti-γ-H2AX | Abcam | Cat#AB11175 |
| Rabbit polyclonal anti-P53 | Proteintech | Cat#10442-1-AP |
| Rabbit monoclonal anti-P21 | Abcam | Cat#AB109199 |
| Mouse monoclonal anti-Actin | ZSGB-BIO | Cat#TA-09 |
| Rabbit polyclonal anti-Temodulin | Abcam | Cat#AB203676 |
| Rabbit monoclonal anti-Tenascin-C | Abcam | Cat#AB108930 |
| Mouse monoclonal anti-Tenascin-C | Thermo Fisher Scientific | Cat#MA5-16086 |
| Mouse monoclonal anti-IL-6 | Abcam | Cat#AB9324 |
| Rabbit polyclonal anti-IL-1β | Abcam | Cat#AB2105 |
| Rabbit monoclonal anti-CD146 | Abcam | Cat#AB75769 |
| Mouse monoclonal anti-CD146 | Novus biologicals | Cat#NBP1-43346 |
| Mouse monoclonal anti-Brdu | Ebioscience | Cat#MA3-071 |
| Mouse monoclonal anti-Vinculin | Proteintech | Cat#66305-1-lg |
| Mouse monoclonal anti-Fmod | Proteintech | Cat#60108-1-lg |
| Rabbit monoclonal anti-P65 | Cell Signaling Technology | Cat#8242 |
| Rabbit monoclonal anti-p-p65 | Cell Signaling Technology | Cat#3033 |
| Rabbit monoclonal anti-p-IKBα | Cell Signaling Technology | Cat#2859S |
| Rabbit monoclonal anti-IKBα | Cell Signaling Technology | Cat#4812S |
| Mouse monoclonal anti-GADPH | Proteintech | Cat#60004-1-lg |
| Rabbit monoclonal anti-p-S6 | Cell Signaling Technology | Cat#4858 |
| Mouse monoclonal anti-S6 | Cell Signaling Technology | Cat#2317 |
| Rabbit polyclonal anti-ATG7 | Proteintech | Cat#10088-2-AP |
| Rabbit monoclonal anti-FABP4 | Abcam | Cat#AB92501 |
| Rabbit polyclonal anti-Col1 | Proteintech | Cat#14695-1-AP |
| Rabbit monoclonal anti-MMP9 | Abcam | Cat#AB76003 |
| Rabbit polyclonal anti-Scx | Abcam | Cat#AB58655 |
| Rabbit polyclonal anti-Mkx | Abcam | Cat#AB66939 |
| Rat monoclonal Anti-CD68 | Ebioscience | Cat#14-0681-82 |
| Rabbit monoclonal anti-α-SMA | Abcam | Cat#AB124964 |
| Rabbit polyclonal anti-P62 | Proteintech | Cat#18420-1-AP |
| Rabbit monoclonal anti-LC3 I/II | Cell Signaling Technology | Cat#12741 |
| Phalloidin-FITC（F-actin） | Solarbio | Cat#CA1620 |
| Goat anti-Mouse IgG(H+L) Cross-Adsorbed Secondary Antibody Alexa Fluor 488 | Thermo Fisher Scientific | Cat#A-11001 |
| Goat anti-Rabbit IgG(H+L) Cross-Adsorbed Secondary Antibody Alexa Fluor 488 | Thermo Fisher Scientific | Cat#A-11008 |
| Goat anti-Rabbit IgG(H+L) Cross-Adsorbed Secondary Antibody Alexa Fluor 594 | Thermo Fisher Scientific | Cat#A32740 |
| Donkey anti-Mouse IgG(H+L) Highly Cross-Adsorbed Secondary Antibody,Alexa Fluor Plus594 | Thermo Fisher Scientific | Cat#A32744 |

**Table S3.** List of chemicals and recombinant proteins used in the study.

| **Chemicals, peptides, and recombinant proteins** | | |
| --- | --- | --- |
| Recombinant rat GDF-5 | RD systems | Cat#853-G5 |
| Recombinant rat TGF-β1 | Peprotech | Cat#AF-100-21C |
| Insulin | Sigma-Aldrich | Cat#10516 |
| Prim-O-glucosylcimifugin | Topscience | Cat#80681-45-4 |
| Recombinant rat TNF-α | Peprotech | Cat#400-14 |
| Recombinant human TNF-α | Peprotech | Cat#400-14 |
| 3-Methyladenine | Selleck | Cat#S2767 |
| Penicillin-Streptomycin | Thermo Fisher Scientific | Cat#15070063 |
| Trypsin-EDTA | Hyclone | Cat#SH30042.01 |
| TRIzol Reagent | Thermo Fisher Scientific | Cat#15596026 |
| Collagenase Type I | Thermo Fisher Scientific | Cat#17100017 |
| Matrigel | Corning | Cat#354234 |
| Crystal Violet Stain solution | Solarbio | Cat#G1062 |
| Collagen I, Rat Tail | Corning | Cat#54236 |
| RNeasy mini Kit | Qiagen | Cat#74104 |
| bFGF2 | Peprotech | Cat#400-29 |
| Recombinant rat EGF | Peprotech | Cat#400-25 |
| Mounting Medium with DAPI | ZSGB-BIO | Cat#ZLI-9557 |
| Dialysis Bag | Solarbio | Cat#YA1077 |
| Dispase | Roche | Cat#10269638001 |
| 1,1,1,3,3,3-hexafluoro-2-propanol solvent | Sigma-Aldrich | Cat#920661 |
| 1-ethyl3-(3-dimehylaminopropyl) carbodiimide hydrochloride | Sigma-Aldrich | Cat#25952-53-8 |
| RIPA Buffer | Thermo Fisher Scientific | Cat#89900 |
| L-Glutamine | Thermo Fisher Scientific | Cat#25030081 |
| L-Ascorbic acid | Sigma-Aldrich | Cat#A5960 |
| SYBR Green Supermix | Thermo Fisher Scientific | Cat#4385612 |
| Dialysis bag | Solarbio | Cat#YA1077 |
| Fetal bovine serum（FBS） | Thermo Fisher Scientific | Cat#10099-141 |
| Osmium tetroxide | Polysciences | Cat#23311-10 |
| DMEM | Hyclone | Cat#SH30021.01B |
| Dexamethasone | Sigma-Aldrich | Cat#D8893 |
| Lyso-Tracker Red probe | Beyotime | Cat# C1046 |
| PLGA | Sigma-Aldrich | Cat#764787 |
| PLGA-PEG | RuiXi Biological Technology | R-PL1003-50K |
| Ultra-low attachment surface | Corning | Cat#3474 |
| TBS | Solarbio | T1080 |
| Tween | Sigma-Aldrich | P9416 |
| **Critical commercial assays** | | |
| ReverTra Ace qPCR RT Kit | TOYOBO | Cat#FSQ-101 |
| Oil Red O stain Kit | Solarbio | Cat#G1262 |
| Senescence β-Galactosidase Staining Kit | Cell Signaling Technology | Cat#9860 |
| Picro Sirius Red Stain Kit | Abcam | Cat#AB150681 |
| Masson’s Trichrome Stain Kit | Solarbio | Cat#G1340 |
| Pierce BCA protein assay Kit | Thermo Fisher Scientific | Cat#23225 |
| Enhanced Chemiluminescence Western Blotting Detection Kit | Thermo Fisher Scientific | Cat#34577 |

**Table S4.** List of primers used in the study.

| **Gene** | **Assay** | **Forward (5’-3’)** | **Reverse (5’-3’)** |
| --- | --- | --- | --- |
| r*Gapdh* | qRT-PCR | GTTCCAGTATGACTCTACCCACG | CATTTGATGTTAGCGGGATCTCG |
| r*Sox2* | qRT-PCR | GGGTTCTTGCTGGGTTTTGATTC | TCTTGCCAGTACTTGCTCTCATG |
| r*Oct4* | qRT-PCR | CGAGAACCTTCAGGAGATATGCA | GCAGAAACATGTTCTCCAGGTTC |
| r*Il-6* | qRT-PCR | GAAGATTCCAAAGATGTAGCCGC | TTACATGTCTCCTTTCTCAGGGC |
| r*Tnf*-α | qRT-PCR | GAGGGAGAGAAGCAACTACAGAC | GTGGGTCAGTATGTGAGAGGAAG |
| r*Il1-β* | qRT-PCR | ACAGTGAGGAGAATGACCTGTTC | CTTCCTGAAGCTCTTGTCGAGAT |
| r*Cxcl1* | qRT-PCR | GTGCAGTTTAAAGATGGTAGGCG | CACCCTAACACAAAACACGATCC |
| hGAPDH | qRT-PCR | TCGGAGTCAACGGATTTGGT | TTGCCATGGGTGGAATCATA |
| hMCP-1 | qRT-PCR | GGCGAATCAGAAGCAGCAAGCAAC | ATTGGCCAGCTGCCGTGTGAA |
| hIL-6 | qRT-PCR | ACTCACCTCTTCAGAACGAATTG | CCATCTTTGGAAGGTTCAGGTTG |
| hIL1-β | qRT-PCR | GGATGCCAGGAAAGGTTCTG | CCAGGTGTGGAGTTCCTGATGT |

**Table S5.** Software and Algorithms.

| Gene ontology | https://geneontology.org/ |
| --- | --- |
| DELPS | This paper |
| GSEA 3.0 | http://software.broadinstitute.org/gsea/index.jsp |
| μCT Evaluation CTAn software | <https://www.blut->scientific.com/bruker-micro-ct-software/ |
| Graph Pad Prism 8.0 | https://www.graphpad.com/ |
| Horos | https://www.horosproject.org/ |
| Venny 2.1 | http://bioinfogp.cnb.csic.es/tools/venny/ |
| Nanoscope analysis 1.9 | https://www.bruker.com/ |
| ImageJ/Fiji | http://fiji.sc |
